# Application of Synchrotron Radiation Based X‐Ray Diffraction in Zeolite Research: Advanced Analysis from Atomic Structure to Dynamic Behavior

**DOI:** 10.1002/advs.202515141

**Published:** 2025-11-14

**Authors:** Weihua Wang, Chenxin Gong, Mingming Chen, Nan Zhang, Jingqing Zhang, Jing Shi, Hongxing Liu, Jiawei Teng, Jianqiang Wang, Yongfeng Hu

**Affiliations:** ^1^ State Key Laboratory of Green Chemical Engineering and Industrial Catalysis Sinopec Shanghai Research Institute of Petrochemical Technology Shanghai 201208 China; ^2^ UNILAB State Key Laboratory of Green Chemical Engineering and Industrial Catalysis East China University of Science and Technology Shanghai 200237 China

**Keywords:** AI‐assisted analysis, atomic structures, dynamic behaviors, synchrotron radiation X‐ray diffraction (SR‐XRD), zeolites

## Abstract

Zeolites, as crystalline materials with regular pore channels, are widely utilized in energy, environmental, and advanced manufacturing sectors. Characterizing zeolites is crucial for understanding their structure and properties, which are essential for various applications. Synchrotron Radiation‐based X‐ray Diffraction (SR‐XRD) has become an advanced tool in zeolite research, providing higher resolution, faster scans, and more precise structural information than laboratory X‐ray diffraction methods. This technique allows for detailed studies, from atomic structures to dynamic behaviors, particularly in understanding structural evolution during synthesis and monitoring changes in the framework during reactions. Moreover, SR‐XRD has made significant contributions to catalytic research by revealing structural alterations during catalytic processes and identifying active sites. However, SR‐XRD still faces challenges in data interpretation and other technological limitations. To overcome these, integrating SR‐XRD with other techniques and using AI‐assisted analysis are expected to further advance zeolite characterization and catalytic research.

## Graphical abstract

Synchrotron Radiation‐based X‐ray Diffraction (SR‐XRD) offers high‐resolution insights and real‐time monitoring of zeolite synthesis, structural transformations, and catalytic reactions, advancing zeolite‐based catalyst development. However, challenges such as data interpretation and technological limitations remain. Combining SR‐XRD with other techniques and AI‐assisted analysis can overcome these issues and accelerate research in zeolite and catalytic studies.

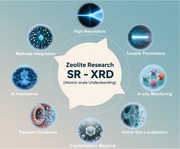

## 1 Introduction

The increasing demand for sustainable energy, environmental protection, and resource‐efficient technologies represents some of the most pressing global challenges of the 21st century.^[^ ^1^, ^2^ ^]^ Tackling these challenges requires the development of advanced materials capable of catalyzing chemical transformations, capturing pollutants, and enabling selective molecular separations. Among the wide range of functional materials, zeolites (crystalline microporous aluminosilicates) have emerged as key platforms due to their unique combination of structural regularity, chemical tunability, and thermal stability.^[^ ^3^, ^4^, ^5^ ^]^

Zeolites are characterized by their atomically precise, 3D pore networks, which provide size‐ and shape‐selective properties, high surface area, and tunable acidity. These features make zeolites essential for a wide range of applications, including catalytic cracking, methanol‐to‐olefin conversions, gas purification, and environmental remediation.^[^ ^6^, ^7^, ^8^, ^9^, ^10^, ^11^ ^]^ The performance of zeolites in these processes is closely related to their atomic structure, which includes framework topology, defect distribution, active site configuration, and dynamic behavior under operating conditions.^[^ ^12^, ^13^ ^]^ Therefore, the rational design, synthesis, and optimization of zeolite materials require highly accurate and comprehensive structural characterization.

The optimization of zeolite performance relies heavily on structural analysis techniques. X‐ray diffraction (XRD) has long been the primary method for determining the structures of zeolites. Laboratory‐based XRD systems using Cu Kα radiation have facilitated the discovery and basic structural characterization of many zeolite frameworks.^[^ ^14^, ^15^, ^16^ ^]^ However, laboratory XRD methods have certain limitations. The monochromaticity of Cu Kα radiation makes it difficult to analyze multi‐level pore structures, such as mesopore‐micropore composite systems. Additionally, the small size (<100 nm) of nano‐crystals and the presence of defects lead to peak broadening and intensity distortion in diffraction patterns, particularly when analyzing submicron‐sized phosphorus‐aluminum zeolites (e.g., DNL‐11).^[^ ^17^ ^]^ It is also difficult to utilize the fixed‐energy light source in analyzing complex systems, such as those with multi‐level pores or metal doping. The peak broadening in nano‐crystals (<100 nm) causes deviations in unit cell parameters, and there is insufficient sensitivity to detect light elements (Si, O) and low‐loading metals (<5 wt%). This limits the ability to locate the local environment of active sites, such as isolated Si atoms in SAPO‐35.^[^ ^18^ ^]^ The static measurement mode (room temperature, ambient pressure) and low time resolution (>10 s) cannot track dynamic processes. For instance, during the CO_2_ adsorption and release, the structure and metal ion environment of LTA zeolite may change rapidly, a process difficult to capture using laboratory XRD.^[^ ^19^ ^]^ These limitations significantly hinder the ability of laboratory XRD in capturing the complex, multi‐scale, and transient structural phenomena that are critical to zeolite functionality.

The introduction of synchrotron radiation‐based X‐ray diffraction (SR‐XRD) has significantly advanced zeolite characterization. SR‐XRD provides much higher brightness, tunable energy across a broad spectral range, superior angular resolution, and millisecond‐scale time resolution (**Table** **1**). This allows for the collection of high‐quality structural data under both ex situ and in situ conditions.^[^ ^20^, ^21^ ^]^ By overcoming the limitations of laboratory XRD, SR‐XRD enables atomic‐level analysis of framework ordering, active site placement, guest‐host interactions, and structural dynamics during synthesis and catalytic reactions. It also allows for the monitoring of crystallization pathways, detection of subtle phase transitions, analysis of hierarchical pore structures and dynamic catalytic processes, and visualization of framework deformation caused by reactant adsorption or catalyst aging.^[^ ^19^, ^20^ ^]^ These capabilities are essential for guiding the design of next‐generation zeolitic materials.

**Table 1:** Comparison of SR‐XRD and Laboratory XRD Parameters.

Technical specifications	Laboratory XRD	SR‐XRD	Performance improvement factor
Light source brightness	10^12^ photons s^−1^	10^18^ photons s^−1^	6 orders
Energy tunable range	Fixed (e.g., Cu/Kα)	0.1‐100 keV	Continuously tunable
Angular resolution	0.02–0.05°	0.001–0.01°	1 order
Time resolution	Minute‐level	Millisecond‐level	3 orders

It is worth noting that XRD, small‐angle X‐ray scattering (SAXS), and wide‐angle X‐ray scattering (WAXS) are all based on Bragg's law (2dsinθ = nλ). From the perspective of the measurement angle range, XRD is a general term for the technique that uses X‐rays to analyze the structure of crystalline or non‐crystalline materials through diffraction/scattering phenomena. The detection angle range is not limited and can be adjusted according to the research purpose. WAXS is primarily used to analyze the long‐range ordered structure of crystals, specifically referring to XRD measurements with larger scattering angles (typically 2θ > 5°). In most cases, when people talk about studying the phases and structures of crystalline materials, they refer to it as XRD. When discussing the microstructure and ordering of soft materials or nanomaterials, especially in the context of SAXS, they are more likely to refer to it as WAXS. **Table** **2** shows X‐ray scattering/diffraction methods in the context of studying zeolites. Therefore, in the field of synchrotron radiation, both WAXS and XRD are generally regarded as diffraction‐based techniques, with WAXS also considered as a type of XRD.

**Table 2:** X‐ray scattering/diffraction methods in the context of studying zeolites.

Techniques	XRD	SAXS	WAXS
Range of 2θ	0°90°, extended to over 150 ° (depending on the sample and equipment)	0.1°–5 °, or even lower angles	5°–90° or higher (measured within a high angle range)
Detection scale	0.1–1 nm (determined by Bragg's law); 1–1000 nm (Analyze peak width through Xie Le formula)	1–100 nm (determined by q range, expandable); 0.5–1000 nm (Through model fitting)	0.1–1 nm (determined by Bragg's law); 1–1000 nm (Analyze peak width through Xie‐Le formula)
Structure information obtained	Long‐range ordered structure: crystal arrangement, lattice constant, grain size, and other information	Nanoscale structure: particle size, pore size distribution, aggregation state (without long‐range ordering requirements)	Atomic level structure: lattice symmetry, interplanar spacing, phase transition, lattice strain (requiring long‐range ordering)

**Figure 1:**
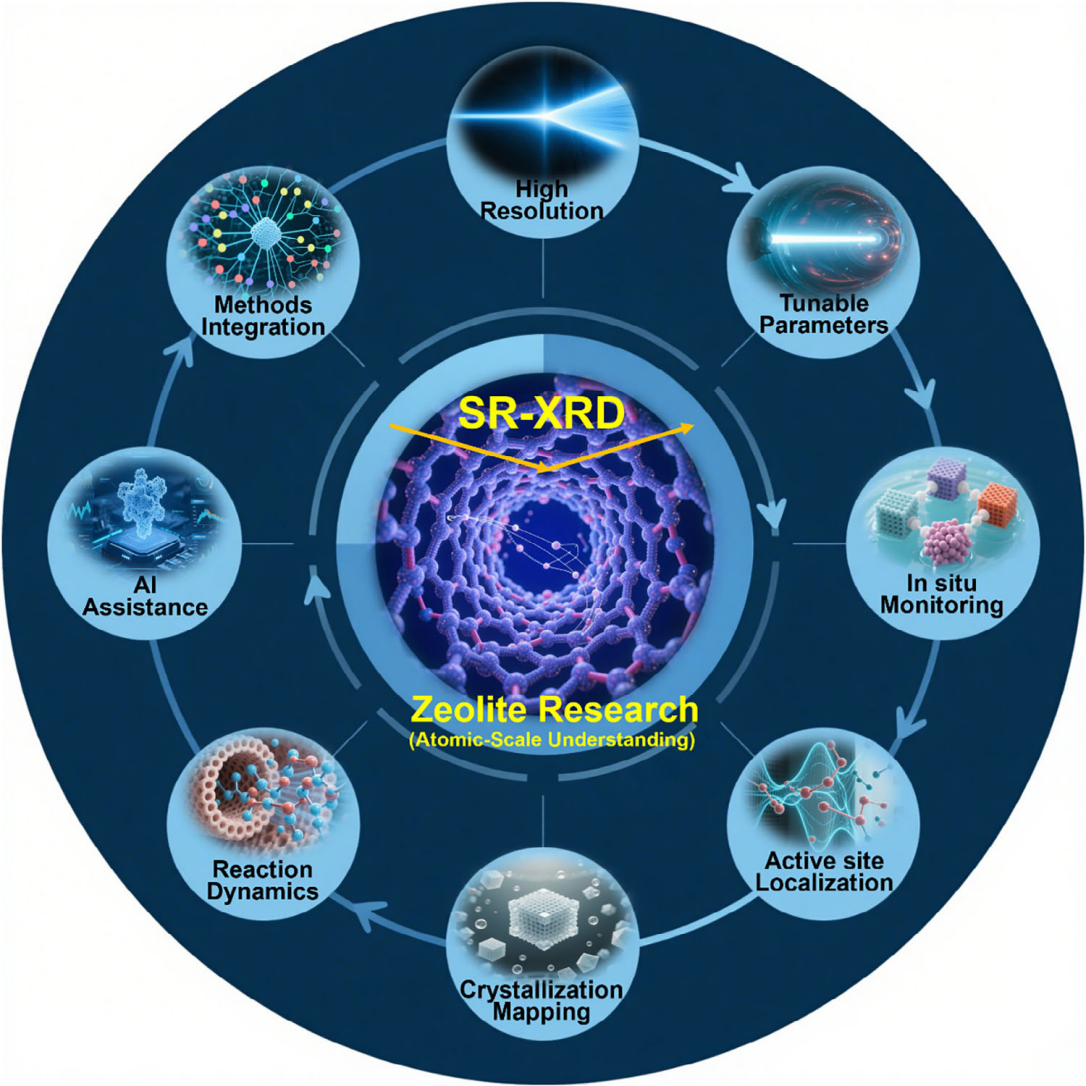
Schematic illustration of SR‐XRD in characterization of zeolite.

In this review, we provide a comprehensive overview of SR‐XRD in zeolite research (**Figure** **1**). We begin by introducing the core principles and technical advancements of SR‐XRD, highlighting why it is well‐suited for complex material systems. We then discuss its role in high‐resolution structural analysis, focusing on key breakthroughs in framework refinement, active site localization, and defect characterization. Next, we explore the capabilities of in situ SR‐XRD for real‐time monitoring of zeolite synthesis, transformation, and post‐synthetic modifications. We also cover in situ SR‐XRD studies, which reveal structural changes during catalytic reactions and adsorption processes. Finally, we examine emerging trends at the intersection of synchrotron science and artificial intelligence, with an emphasis on machine learning‐assisted diffraction analysis and its potential to accelerate zeolite discovery and optimization. This review aims to demonstrate how SR‐XRD is reshaping our atomic‐level understanding of zeolite materials and advancing their rational development for energy and environmental applications.

## 2 SR‐XRD: Fundamentals and Advancements

### 2.1 Fundamental Principles and Capabilities of SR‐XRD

SR‐XRD is based on Bragg's law (2dsinθ = nλ), which defines the conditions for constructive interference of X‐rays scattered by periodic atomic planes. In contrast to laboratory XRD with fixed wavelength, SR‐XRD employs highly collimated, high‐flux X‐ray beams (shown in Table 1). These beams are produced when relativistic electrons are deflected by strong magnetic fields in a synchrotron storage ring. This fundamental difference gives SR‐XRD unique and powerful capabilities for materials characterization.^[^ ^22^, ^23^, ^24^ ^]^

From Table 1, among the key capabilities of SR‐XRD is its exceptionally high brightness, often 6 orders of magnitude higher than that of laboratory sources.^[^ ^25^, ^26^ ^]^ This enables the study of micro‐ and nano‐scale crystallites with enhanced signal‐to‐noise ratios. The tunable photon energy, spanning a wide spectral range from soft to hard X‐rays, allows for optimization of penetration depth, improved anomalous scattering experiments, and selective elemental contrast near absorption edges.^[^ ^27^ ^]^ According to Bragg's law, energy is inversely proportional to wavelength. High‐energy X‐rays, with the same crystal plane spacing (d), result in smaller θ values, compressing the 2θ range and reducing peak broadening. This allows for a wider data range to be measured in the same amount of time, making it suitable for penetrating thicker samples (such as zeolite in high‐pressure reaction cells). Low‐energy X‐rays lead to larger θ values, expanding the 2θ range and increasing peak spacing, which is beneficial for distinguishing closely spaced crystal planes (e.g., the 100 and 110 planes of zeolite). This difference directly affects the diffraction peak resolution, and SR‐XRD, with its adjustable energy, can cater to various research needs. The superior angular and spatial resolution, achieved through precise beam optics, reduces peak overlap and broadening. As shown in Table 1, the angular resolution of laboratory XRD typically ranges from 0.02° to 0.05°, while SR‐XRD offers much higher angular resolution, typically achieving between 0.001° and 0.01°, representing an improvement by an order of magnitude. This facilitates the detection of subtle lattice distortions, defect states, and complex phase intergrowths in polycrystalline materials.^[^ ^28^ ^]^ Additionally, the use of advanced detectors, such as the Mythen series or high‐speed imaging plates, allows for rapid data acquisition and time‐resolved diffraction studies.^[^ ^29^ ^]^ These studies can achieve temporal resolution on the millisecond scale, enabling the observation of transient phenomena during synthesis and catalytic reactions.^[^ ^30^, ^31^ ^]^

**Figure 2:**
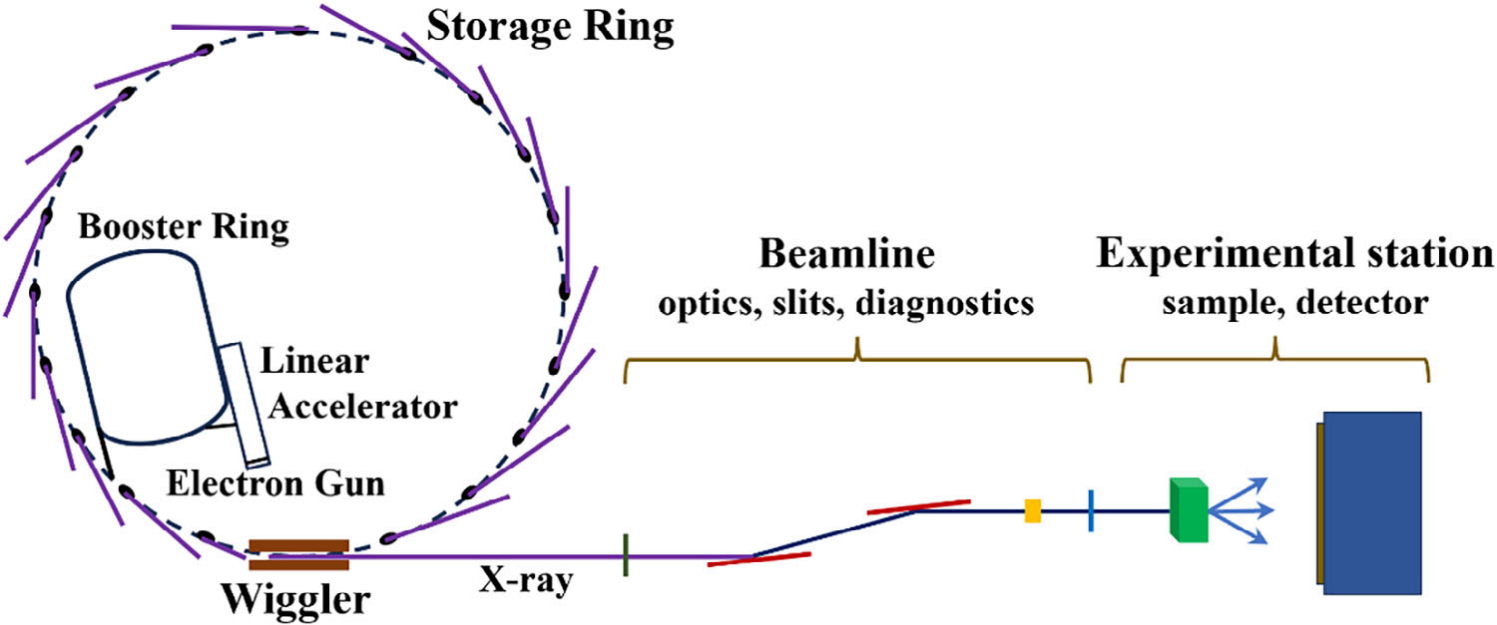
Schematic diagram of the components of synchrotron radiation.

A typical SR‐XRD experimental setup consists of a synchrotron storage ring, monochromators (e.g., Si (111) double crystals), focusing mirrors, beam slits for spatial shaping, and fast detectors designed for optimal angular coverage and sensitivity (**Figure** **2**).^[^ ^32^, ^33^, ^34^ ^]^ The beamline optics collimate and focus the selected monochromatic X‐ray beam onto the sample, which is usually mounted in capillaries or in situ reaction cells. Controlled sample rotation or oscillation minimizes preferred orientation effects, ensuring the collection of comprehensive diffraction data.

These features make SR‐XRD particularly well‐suited for the advanced characterization of complex materials like zeolites. The technique allows for precise determination of framework structures, atomic site occupancies, and long‐range order. It also provides insights into dynamic processes, such as phase transitions, structural flexibility, and framework degradation under operational conditions. As a result, SR‐XRD has become an essential tool in the rational design and optimization of functional porous materials.^[^ ^35^, ^36^ ^]^

### 2.2 Factors Promoting the Progress and Development of Synchrotron Radiation

The advancement of SR‐XRD from a static structural probe to a dynamic monitoring tool marks a significant breakthrough in materials science. Recent progress in in situ SR‐XRD techniques has made it possible to observe structural changes in real‐time during synthesis, phase transformations, catalytic reactions, and material degradation under realistic conditions.^[^ ^23^, ^37^, ^38^, ^39^, ^40^ ^]^

A significant technological advancement is the integration of high‐temperature, high‐pressure reaction cells into synchrotron beamlines. These systems enable precise control over environmental parameters, such as temperature (up to 2000 °C), pressure (up to 100 GPa), and gas atmosphere. This capability allows for the simulation of industrially relevant synthesis and reaction conditions. For instance, Zhou et al.^[^ ^23^ ^]^ utilized SR‐XRD to monitor real‐time phase transitions, stress development, and lattice deformation in materials at extreme temperatures up to 1500 °C. Similarly, Thompson et al.^[^ ^37^ ^]^ measured tetragonal Fe_3_S in a laser‐heated diamondanvil cell (LH‐DAC) at 126 GPa and 2500 K using in situ XRD. These examples highlight the crucial role of advanced environmental control in enabling in situ investigations.

Time‐resolved SR‐XRD enhances the technique's capabilities by enabling the observation of fast structural dynamics with high temporal resolution. The use of rapid‐scan detectors, such as the Mythen 2D system, in combination with intense synchrotron sources, allows for the collection of full diffraction profiles at millisecond scale. This development has made it possible to track rapid processes, such as zeolite nucleation, phase transformation kinetics, and adsorbate‐induced framework distortions. In a key study, Losch et al.^[^ ^38^ ^]^ employed in situ SR‐XRD to explore the dynamic structural changes in H‐ZSM‐5 catalysts during the methanol‐to‐olefin (MTO) reaction by precisely tracking changes in unit cell parameters and diffraction peak broadening. The accumulation of hydrocarbon pool species within the zeolite channels induced localized lattice strain, detected as subtle shifts in peak positions, while the progressive broadening of diffraction peaks indicated partial pore blockage and loss of crystallinity. These combined structural and textual changes directly correlated with the observed decline in catalytic activity, demonstrating how hydrocarbon pool‐induced deformation and blockage lead to catalyst deactivation. Their findings revealed how hydrocarbon pool accumulation leads to lattice strain and pore blockage, ultimately causing catalyst deactivation.

SR‐XRD is often used in conjunction with other in situ techniques, such as X‐ray absorption spectroscopy (XAS), SAXS, and infrared (IR) spectroscopy. However, it stands out for its ability to resolve atomic‐level crystallographic changes. Advances in detector technologies, such as 2D fast readout detectors, and the development of multi‐modal in situ cells that integrate XRD with simultaneous spectroscopic measurements, have significantly broadened the scope of in situ analysis. For example, Hu et al.^[^ ^39^ ^]^ used in situ SR‐XRD and SAXS to study the structural evolution of mesoporous silica‐alumina nanospheres during adsorption and hydrophobicity tests, demonstrating the complementary role of scattering and diffraction in dynamic systems. Gatões et al.^[^ ^40^ ^]^ employed in situ SR‐XRD to monitor the real‐time structural changes of steel during tensile deformation, unveiling the microscopic mechanisms underlying plastic deformation and stress concentration. This provided crucial data for understanding and improving the material's mechanical properties.

These technological advancements have significant implications for zeolite science. In situ SR‐XRD allows for the direct observation of crystallization pathways during hydrothermal synthesis, the identification of metastable intermediate phases, and the exploration of structural flexibility under catalytic turnover conditions. By linking static structural data with dynamic functional behavior, SR‐XRD has become an essential tool for understanding the complex relationship between structure, environment, and performance in zeolitic materials.

## 3 Applications of SR‐XRD in Structural Studies of Zeolites

### 3.1 High‐Resolution Structural Analysis of Zeolite Frameworks

Zeolites have complex frameworks made of corner‐sharing TO_4_ tetrahedra (T = Si, Al, etc.). These frameworks define their pore structures and functional properties.^[^ ^6^ ^]^ Accurately resolving these frameworks, especially the atomic positions, pore geometries, and symmetry relationships, is crucial for understanding and optimizing catalytic activity, molecular sieving, and thermal stability. SR‐XRD provides crystallographic insights with atomic‐level precision and allows for high‐quality diffraction pattern acquisition with minimal background noise, excellent peak resolution, and well‐determined peak profile. This capability allows the Rietveld refinement of complex zeolitic structures. It enables precise determination of unit cell parameters, space groups, atomic positions, and pore architectures. The technique is particularly useful for distinguishing similar framework types and identifying small deviations from idealized symmetry.^[^ ^41^, ^42^, ^43^, ^44^, ^45^ ^]^

Van Koningsveld et al.^[^ ^41^ ^]^ demonstrated the use of SR‐XRD to precisely determine the framework structures of various zeolites. They highlighted its ability to resolve detailed structural features, such as aluminum siting and pore dimensionality. With SR‐XRD, isostructural variants were differentiated, and framework distortions that directly influence adsorption and catalytic performance were identified.

**Figure 3:**
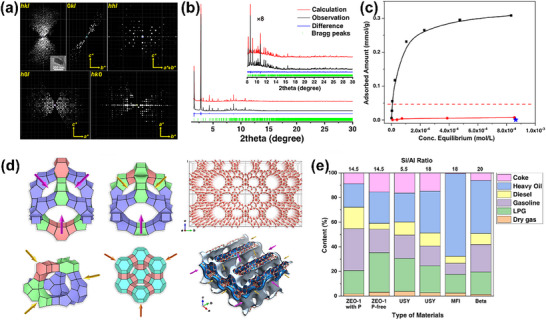
a) 3D and 2D reciprocal lattice reconstructed from a typical cRED dataset. b) ZEO‐1 SR‐XRD and Refinement Patterns of Molecular Sieves; c) Nile Red adsorption isotherms (equilibrium concentration vs adsorbed amount); d) The three supercages and channel system in ZEO‐1; (e) Catalytic performance of several zeolite catalysts in the conversion of heavy oils changing Si/Al ratios. Reproduced with permission.^[^ ^42^ ^]^ Copyright 2021, The American Association for the Advancement of Science.

Building on these capabilities, Lin et al.^[^ ^42^ ^]^ utilized SR‐XRD combined with electron diffraction to determine the crystal structure of ZEO‐1, a novel aluminosilicate zeolite (**Figure** **3**). SR‐XRD provided detailed long‐range crystallographic information, allowing the determination of the zeolite's framework topology, unit cell parameters, and pore geometry. It confirmed the presence of intersecting extra‐large pores with precise structural details. On the other hand, electron diffraction (specifically cRED) was employed to analyze short‐range structures, revealing fine details of atomic arrangements in the nanoscale regions and identifying localized structural defects or variations that SR‐XRD could not detect. ZEO‐1 features a unique 3D pore system formed by 16×12‐membered rings. The obtained structural data revealed a highly ordered framework, free from aluminum clustering. This underscores the potential of ZEO‐1 in catalytic and separation applications for bulky molecules.

**Figure 4:**
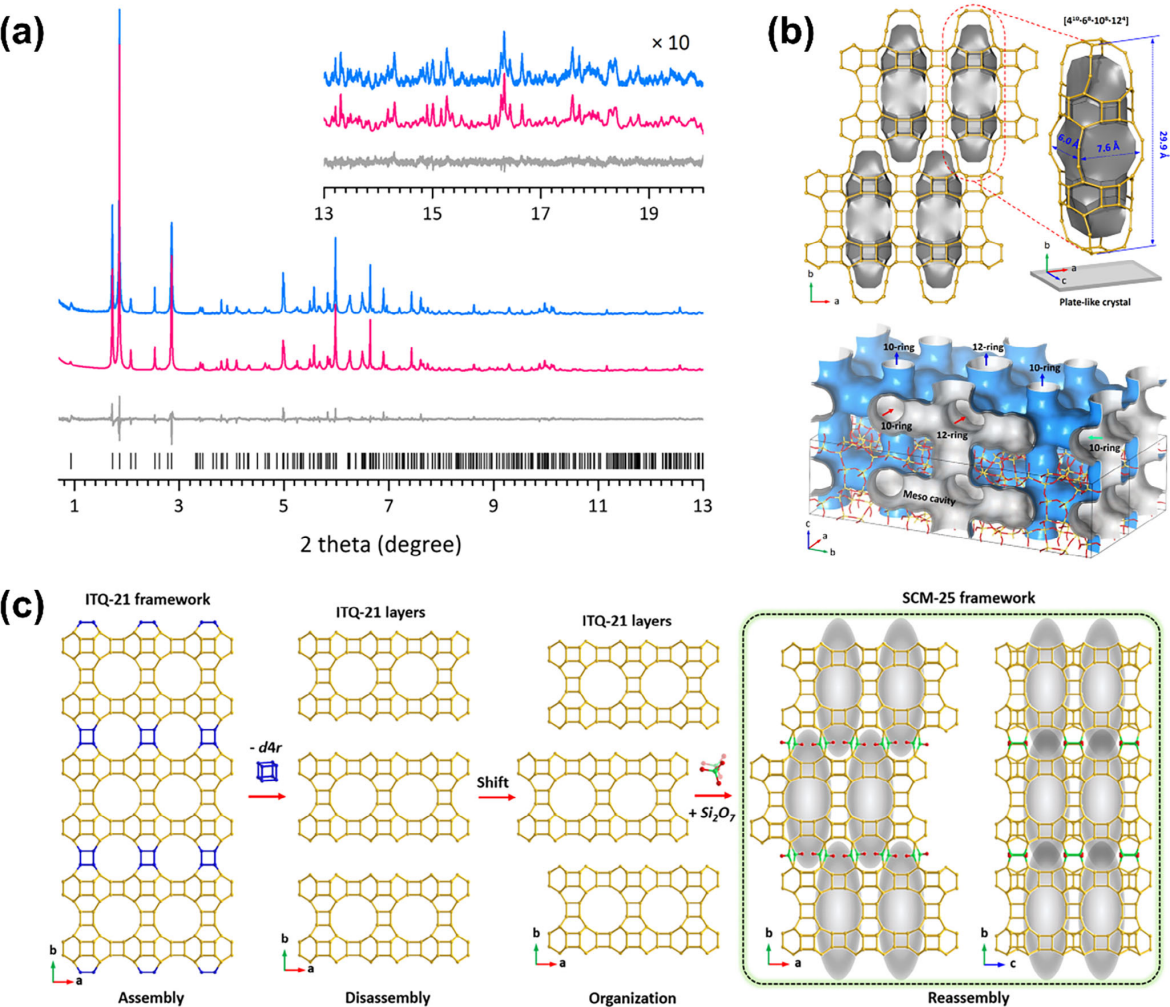
a) SR‐XRD pattern and Rietveld refinement profile of SCM‐25 (λ=0.412836 Å); b) Nature tile of the meso‐cavity and its locations in the SCM‐25 framework and its unique channel system; c) Structural relationship between ITQ‐21 and SCM‐25. Reproduced with permission.^[^ ^45^ ^]^ Copyright 2022, American Chemical Society.

Luo et al.^[^ ^43^, ^44^, ^45^ ^]^ made further advances by combining SR‐XRD with continuous rotation electron diffraction (cRED) to determine the frameworks of SCM‐14, SCM‐15, and SCM‐25 zeolites. SR‐XRD provided detailed long‐range crystallographic data, allowing for the precise determination of the zeolites' framework structures, unit cell parameters, and the nature of their large pores and interconnected channels. Specifically, SR‐XRD was essential in confirming the overall framework and long‐range ordering, including the 12×12×10‐ring channels in SCM‐15 and the meso‐cavities in SCM‐25. On the other hand, electron diffraction, particularly 3D electron diffraction (3D‐ED), was used to analyze the finer details of these zeolites' local structures. It was crucial in resolving the atomic arrangements in smaller crystals, especially for SCM‐25, where it helped to reveal the ordered meso‐cavities and the connectivity of the 12×12×10‐ring channels. These features are crucial for improving mass transport and catalytic accessibility (**Figure** **4**).

These studies collectively demonstrate that SR‐XRD is not just a tool for static structure determination. It can be combined with electron diffraction, serving as a gateway for discovering new framework topologies, evaluating framework defects, and linking atomic‐scale structures to functional performance.^[^ ^41^, ^42^, ^43^, ^44^, ^45^ ^]^ The ability to accurately map pore architectures and framework flexibility is key to the rational design of next‐generation zeolites with tailored properties for catalysis, adsorption, and separation applications.

### 3.2 In Situ Monitoring of Synthesis and Framework Evolution

While static structural information is critical, understanding the dynamic evolution of zeolites during synthesis and under reaction conditions is equally vital. In situ SR‐XRD has emerged as a transformative tool for capturing the real‐time structural evolution of zeolites, shedding light on crystallization pathways, pore development, and framework stability under realistic environments.^[^ ^46^, ^47^, ^48^, ^49^, ^50^, ^51^, ^52^, ^53^, ^54^, ^55^, ^56^, ^57^ ^]^

#### 3.2.1 Real‐Time Tracking of Crystallization and Phase Formation

In situ SR‐XRD enables the continuous observation of zeolite formation from amorphous precursors during hydrothermal synthesis.^[^ ^46^, ^47^, ^48^, ^49^ ^]^ The WAXS used in this section is a wide‐angle application of SR‐XRD, with the same core principles as XRD, primarily used to analyze the long‐range ordered structure of crystals. SAXS combined with WAXS is used to analyze the evolution of nanoscale particles and pores, capturing the short‐range ordered structural changes in the early stages of zeolite synthesis. By collecting diffraction patterns at short time intervals, it reveals the onset of nucleation, growth kinetics, phase competition, and metastable intermediate states. Zeolite crystallization typically follows an induction period, with rapid nucleation and growth. Distinct phase transitions are observed through changes in diffraction peak positions and intensities. These insights help fine‐tune synthetic parameters, promoting the formation of desired frameworks while suppressing unwanted phases, thus optimizing yield and material quality.

**Figure 5:**
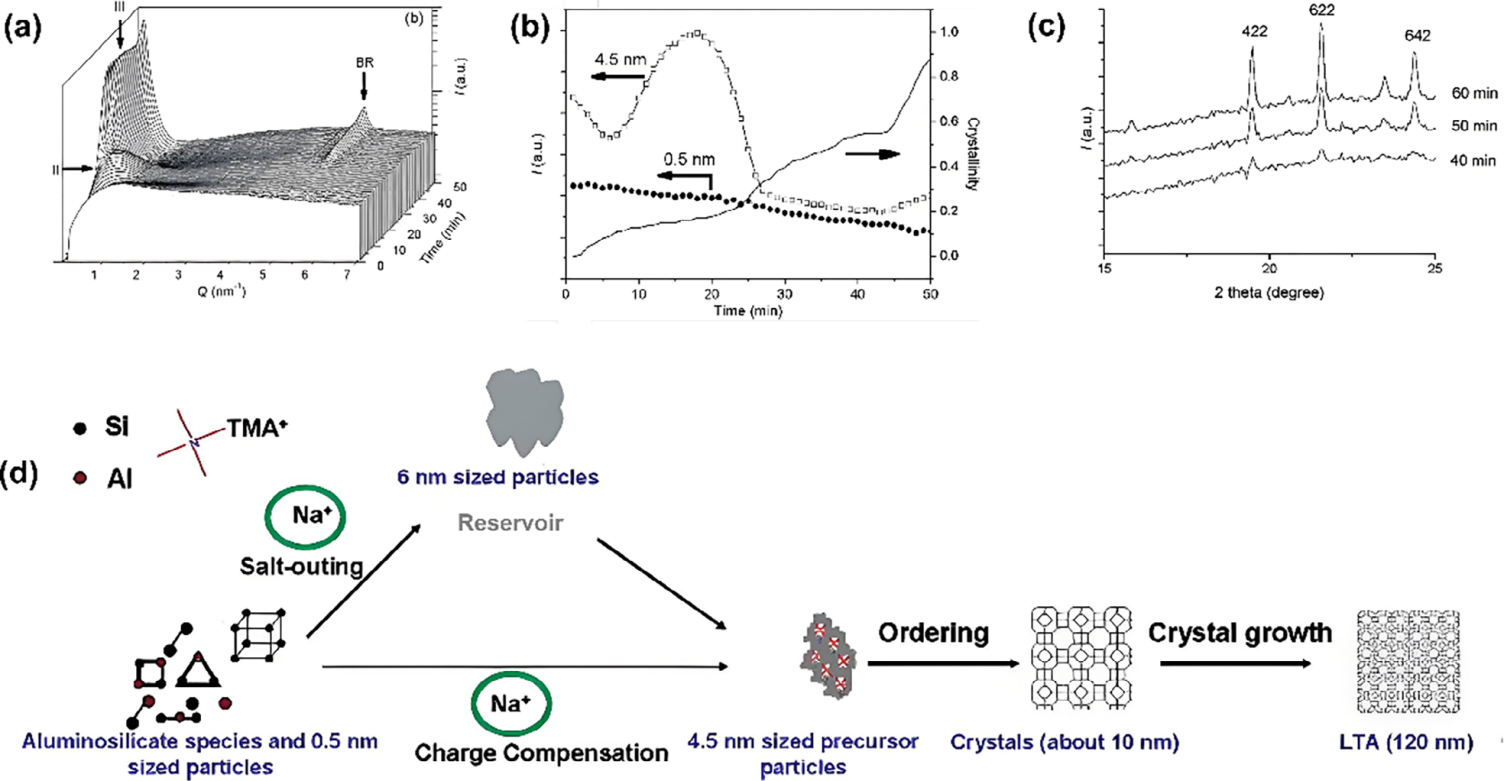
a) Time‐resolved SAXS patterns of the synthesis solution. b) Corresponding to the scattering intensity changes of primary units, secondary particles, and Bragg reflections. c) WAXS patterns of the synthesis solution at different crystallization times. (λ=1.4000 Å) d) The proposed crystallization mechanism. Reproduced with permission.^[^ ^46^ ^]^ Copyright 2007, American Chemical Society.

Fan et al.^[^ ^46^ ^]^ used WAXS combined SAXS to monitor real‐time structural changes during the synthesis of nanosized zeolite A (**Figure** **5**). SAXS provided information about the structure of the zeolite at the nanoscale, revealing changes in the size and shape of the zeolite particles during the early stages of crystal growth. WAXS, on the other hand, provided higher‐angle structural data, confirming the arrangement and order of the zeolite crystals. By combining these two techniques, the researchers were able to gain a deeper understanding of the transition from disorder to order in the zeolite and uncover key factors influencing crystal growth.

Tompsett et al.^[^ ^47^ ^]^ used combined in situ WAXS and SAXS techniques to study the structural evolution of silicon zeolite during microwave synthesis, tracking its transition from solution to solid. The focus was on nucleation, crystal growth, and structural changes during synthesis. In situ WAXS, by observing high‐angle diffraction data, confirmed the ordered structure and lattice changes of the zeolite crystals, enhancing the understanding of factors influencing nucleation and crystal growth. SAXS, on the other hand, provided nanoscale structural information about the zeolites during microwave synthesis, revealing the growth and morphological changes of the zeolite particles. Additionally, the effects of microwave irradiation on crystallization were explored, offering key information for optimizing synthesis conditions and improving the properties of silicon zeolites. Panzarella et al.^[^ ^48^ ^]^ employed in situ SAXS/WAXS to study the synthesis process of NaY, NaA, and Beta zeolites under microwave radiation. By analyzing diffraction peaks, WAXS revealed the ordering of zeolite crystals. As the peaks intensified over time, it indicated the transition from disorder to order, helping understand the crystal growth process. SAXS used low‐angle diffraction data to reveal the size, shape, and aggregation state of zeolite particles. The data showed that microwave radiation affected the morphology and size of the particles. The focus of the study was on real‐time monitoring of the nucleation, crystal growth, and structural evolution of zeolites under microwave synthesis conditions. The significance of this research lies in providing important experimental evidence for the optimization of microwave synthesis of zeolite materials.

**Figure 6:**
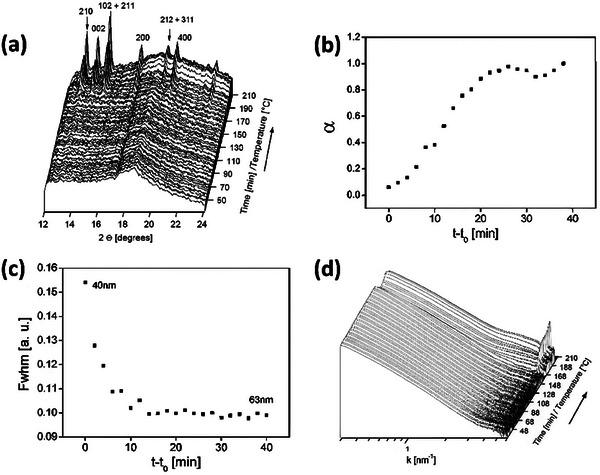
Test results and analysis of the CoAPO‐5 zeolite crystallization process. a) WAXS patterns. b) (100) crystal face variation with time. c) Experimental full‐width at half‐maximum of the same reflection. (d) SAXS patterns. (λ=1.00 Å) Reproduced with permission.^[^ ^49^ ^]^ Copyright 2005, American Chemical Society.

Grandjean et al.^[^ ^49^ ^]^ combined WAXS and SAXS to investigate the crystallization mechanism of CoAPO‐5 zeolites under hydrothermal conditions (**Figure** **6**). During hydrothermal synthesis, the WAXS diffraction peaks of CoAPO‐5 gradually intensified over time, indicating that the crystal structure evolved from a disordered to an ordered state, thus revealing the zeolite crystallization process. Meanwhile, analysis of SAXS low‐angle data showed that particle size increased and morphology gradually transformed from disordered colloidal aggregates to well‐defined nanostructures. By combining these two techniques, it is concluded that CoAPO‐5 crystallization proceeds through the gradual conversion of initial disordered aggregates into ordered crystals under hydrothermal conditions.The examples above emphasized the in situ monitoring of the zeolite synthesis and framework evolution using SR‐XRD combined SAXS technology.^[^ ^46^, ^47^, ^48^, ^49^ ^]^ It underscores the crucial role of synchrotron radiation techniques in tracking the dynamic changes during synthesis, offering valuable insights into the structural evolution and framework stability, which are essential for the design and optimization of advanced catalytic materials.

#### 3.2.2 Insights into Pore Development and Structural Rearrangement

The evolution of the pore system during synthesis and post‐treatment significantly influences the accessibility and diffusion properties of zeolites. SR‐XRD (WAXS), often combined with complementary techniques like SAXS, provides critical information on the development of hierarchical porosity, meso‐ and micropore connectivity, and framework densification.^[^ ^50^, ^51^, ^52^, ^53^ ^]^ In the synthesis of zeolites, XRD is mainly used to determine the crystal structure of the zeolite, helping to identify whether a zeolite with a specific pore structure has been formed. These structures are closely related to the arrangement of pores, and the position and intensity of diffraction peaks in the XRD pattern reflect the orderliness of the pores and crystallinity and further revealing the ordered arrangement of the pores. SAXS provides detailed information about pore size, morphology, and distribution, especially at the nanoscale. By analyzing the position of SAXS peaks, the pore size range can be inferred. SAXS can also analyze data using different models (e.g., Porod model, Guinier model) to obtain pore size distribution. In the early stages of synthesis, SAXS can track the formation process of pores, including nucleation, growth, and distribution changes. If the pores are ordered and uniform, the SAXS pattern will display clear characteristic peaks; conversely, if the pore structure is disordered, the pattern may lack distinct peaks or show broad scattering peaks. By combining XRD and SAXS, a comprehensive understanding of the formation and distribution of zeolite pores can be achieved.^[^ ^50^, ^51^, ^52^, ^53^ ^]^

The nanostructures of FAU and LTL zeolites were studied using WAXS and SAXS methods by Zholobenko et al.^[^ ^50^ ^]^ confirming the formation process of a regular mesoporous network. WAXS analysis showed that FAU and LTL zeolites each formed highly ordered crystal structures with small crystal sizes during synthesis. SAXS was primarily used to investigate the pore structures of the zeolites, providing information about the pore size and distribution, with the main pore sizes ranging from a few nanometers to tens of nanometers. The formation of these pore structures is closely related to the synthesis conditions, and different synthesis methods may result in variations in pore ordering. By combining the crystallographic information from WAXS and the pore information from SAXS, a deeper understanding of the formation mechanism of the zeolites during synthesis is achieved.

Hriljac ^[^ ^51^ ^]^ used high‐pressure SR‐XRD to monitor in real‐time the changes in the pore structure and framework of zeolites under high pressure. Through the analysis of high‐pressure SR‐XRD data, the study showed significant changes in the crystal structure of zeolites under high‐pressure conditions, particularly in the deformation of channels and framework structures. By analyzing diffraction patterns at different pressures, the study revealed the structural stability and deformation mechanisms of zeolites under high pressure. The study found that under high pressure, the framework structure of zeolites changes, with pore size and shape compressing or deforming as pressure increases. Additionally, different types of zeolites respond differently to pressure, with some maintaining stable structures, while others experience significant structural damage or pore shrinkage. This provides important insights into the stability and pore regulation of zeolites under high‐pressure conditions.

Han et al.^[^ ^52^ ^]^ used XRD to monitor the process of fly ash conversion into high zirconium content zeolite and evaluated its efficiency in removing As(V) from solution. The study showed that as the zirconium content increased, the zeolite's framework and pore structure changed, particularly in terms of pore size and distribution. The high zirconium content zeolite exhibited larger pore volume and improved pore structure, which is crucial for enhancing As(V) adsorption efficiency. This study provides valuable insights for designing and optimizing the synthesis of zeolite materials to efficiently remove water pollutants.

**Figure 7:**
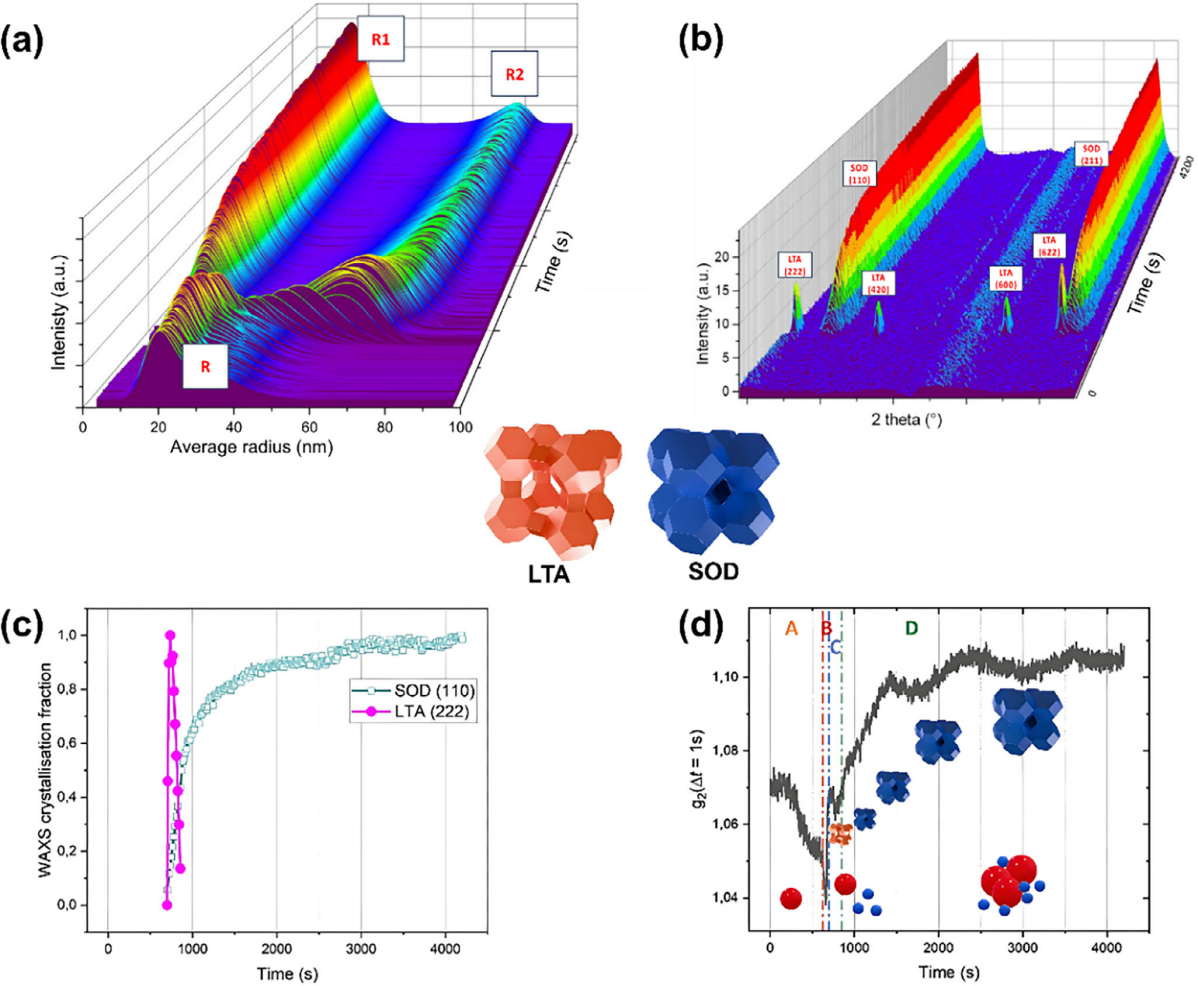
Data analysis obtained during the nanozeolite formation. a) In situ radius distributions of the nanoparticles from the SAXS analysis. b) Intensities in the WAXS range of the zeolite at 363 K. (λ=1.378 Å) (c) Time evolution of the LTA (222) and SOD (110) peaks. d) The analysis of zeolite at various stages of its formation. Reproduced with permission.^[^ ^53^ ^]^ Copyright 2025, Elsevier.

Garcia et al.^[^ ^53^ ^]^ used WAXS combined with in situ SAXS to study the formation dynamics of nano‐zeolite zeolites in real time (**Figure** **7**). By analyzing SAXS data collected at different time points during the synthesis, the study revealed the dynamics of zeolite formation, including the transformation from disordered aggregates to ordered nanoparticles. Additionally, the study showed that by observing the evolution of particle size and morphology, the structural changes and kinetic features at various stages of the synthesis could be captured. WAXS was used to provide structural information at higher angles. WAXS data allowed the researchers to observe changes in the crystallinity and order of the zeolites, especially during the crystallization process. During the synthesis, the pore size and structure change as the reaction progresses. Initially, the pore structure is disordered, but as crystallization occurs, the pores become more ordered, eventually forming a nano‐zeolite with a specific pore structure. Reaction conditions, such as temperature, time, and additives, significantly influence the formation and stability of the pores.

The above examples demonstrate that the SR‐XRD technique enables a detailed examination of the evolution of pore development and structural rearrangements during the synthesis process.^[^ ^50^, ^51^, ^52^, ^53^ ^]^ By tracking the changes in pore size and shape at various stages, they can uncover the mechanisms through which reactants undergo chemical transformations, ultimately forming zeolites with well‐defined pore structures. SR‐XRD study offers valuable insights into the dynamic development of pores and the structural adjustments in nano‐zeolites, providing a theoretical foundation for optimizing synthesis procedures and improving their catalytic performance.

#### 3.2.3 Dynamic Evolution of Framework Structures under Synthesis Conditions

Zeolite frameworks often undergo subtle or substantial structural rearrangements during synthesis, ion exchange, or post‐synthetic modifications.^[^ ^54^, ^55^, ^56^, ^57^ ^]^ In situ SR‐XRD could provide direct evidence of such dynamics, capturing lattice parameter variations, phase transformations, and defect generation or healing processes. Temperature‐ and pressure‐dependent SR‐XRD studies have elucidated phenomena such as framework breathing, contraction, or collapse under steam treatment, as well as the stabilization of metastable phases through kinetic control. By mapping these structural changes over time and varying conditions, researchers gain mechanistic insights into the factors governing zeolite stability, flexibility, and catalytic durability.

Zhou et al.^[^ ^54^ ^]^ used in situ SR‐XRD to investigate the disassembly and reorganization mechanisms of germanosilicate zeolites under the influence of HCl vapor. By using SR‐XRD, the researchers were able to monitor real‐time structural changes in the zeolite crystals, particularly during the disassembly and reorganization processes. The results showed that HCl vapor induced the disassembly of the zeolite, leading to changes in its pore structure, and under certain conditions, the zeolite underwent a reorganization process from disordered to ordered. By analyzing the SR‐XRD data, the researchers were able to track changes in the lattice parameters and diffraction peaks, thereby revealing the impact of HCl vapor on the zeolite structure.

**Figure 8:**
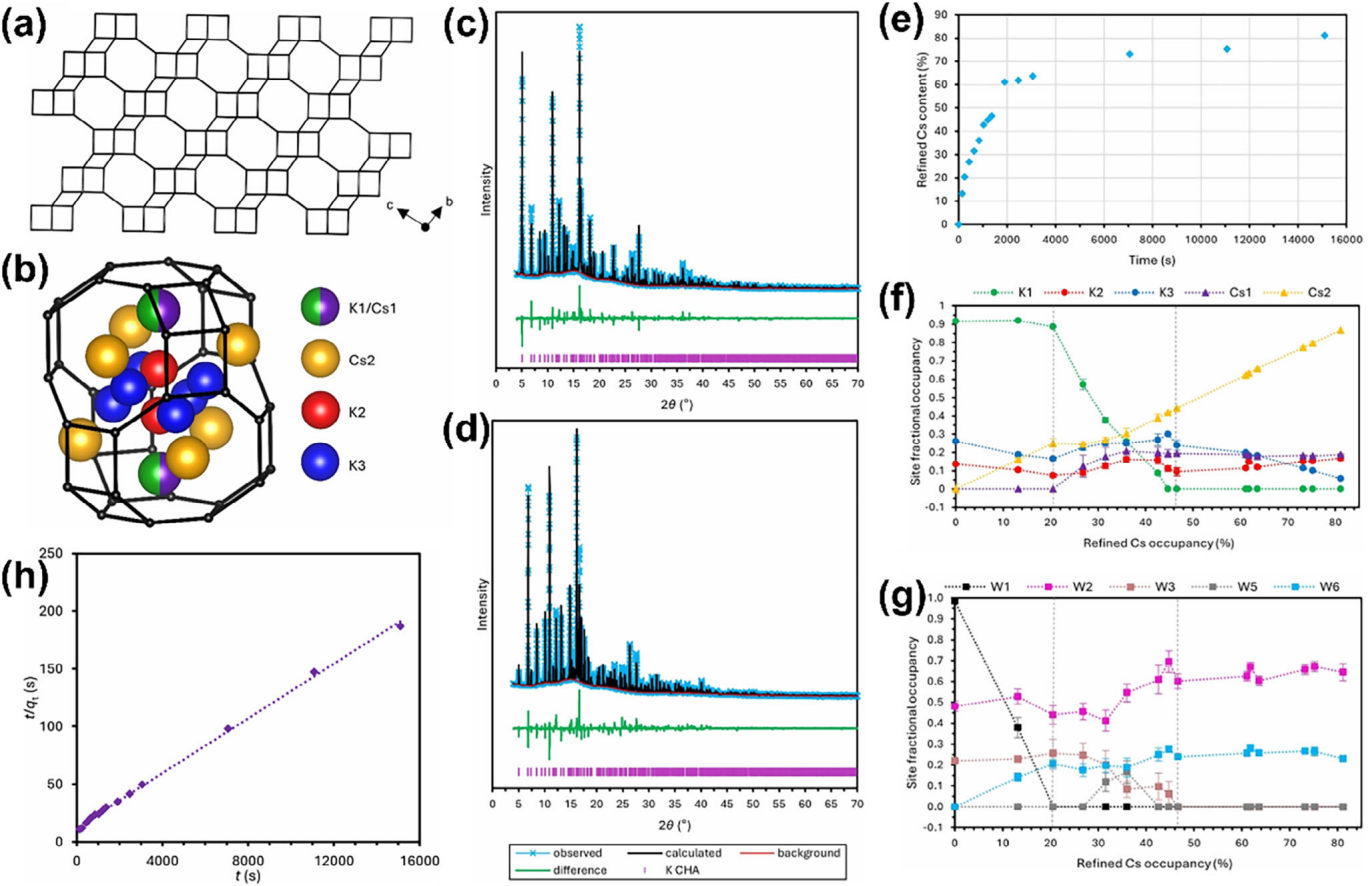
a) Chabazite lattice fragment; b) Depiction of a chabazite cage showing the cation sites; Rietveld refinements of K‐CHA XRD patterns recorded at t = 0 s c) and 15 099 s d); (λ=0.8244 Å) e) Plot of the proportion of occupied extraframework cation sites containing Cs^+^ as a function of time; f) Plot of occupancies for the extraframework cation sites; g) An equivalent plot to (f) for the fractional occupancies on the crystallographic water sites; h) Plot of the linear pseudo‐second‐order kinetic model. Reproduced with permission.^[^ ^55^ ^]^ Copyright 2024, American Chemical Society (CC‐BY 4.0).

Parsons et al.^[^ ^55^ ^]^ employed in situ powder SR‐XRD technology to study the mechanism of cesium ion exchange in chabazite zeolite (**Figure** **8**). By using in situ powder XRD, the real‐time changes in the crystal structure and lattice parameters of the zeolite during the cesium ion exchange process were able to be observed. The results showed that cesium ions gradually replaced sodium ions in the zeolite channels, and this exchange process was accompanied by slight adjustments in the crystal structure, particularly in the pore size. By analyzing the changes in lattice constants and diffraction peaks in the diffraction patterns, the researchers further revealed the kinetic features and structural evolution of the zeolite during the ion exchange process.

**Figure 9:**
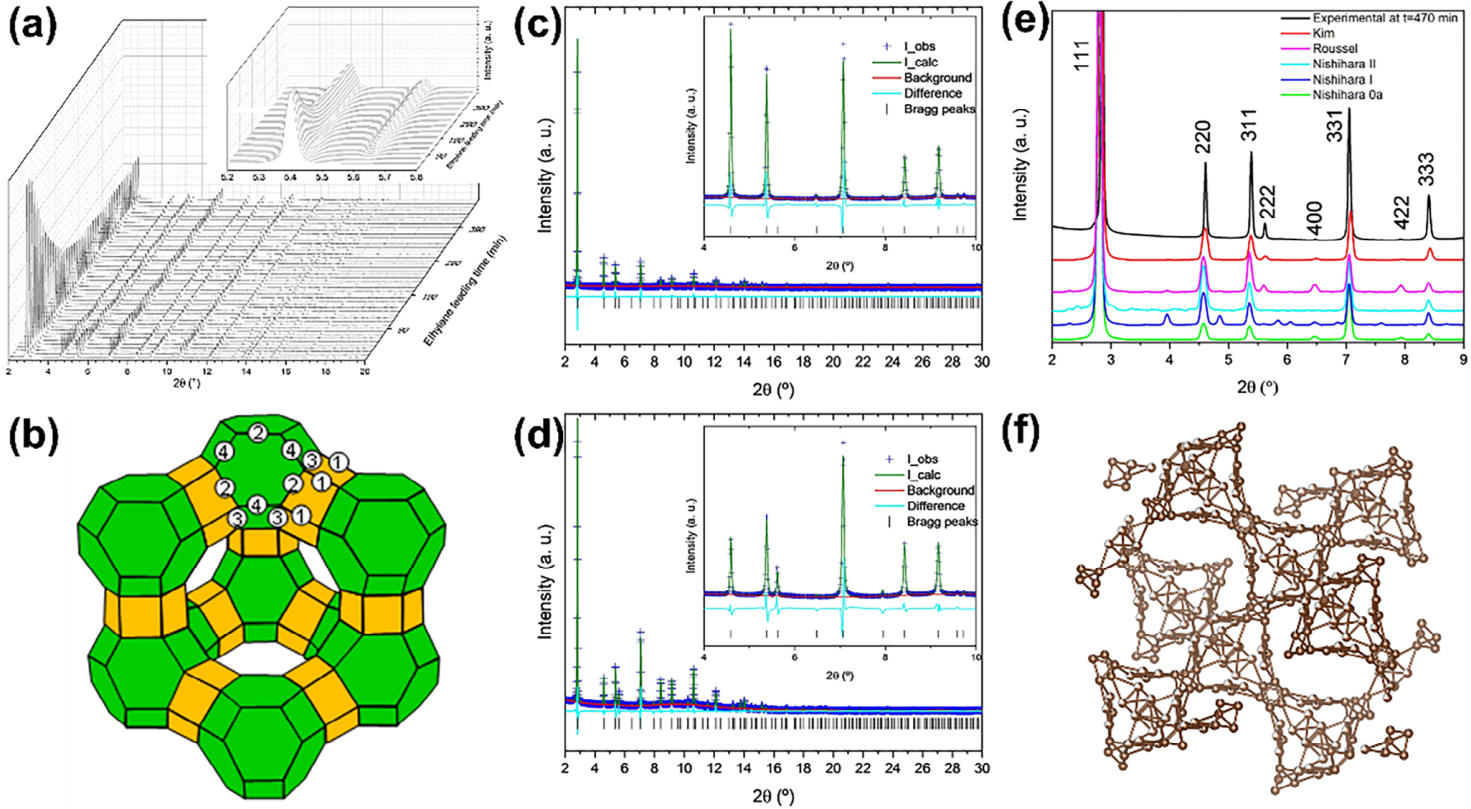
a) Time resolved in situ XRPD patterns of HY zeolite during ZTC formation at 680 °C; (λ=0.6919 Å) b) FAU structure; Rietveld refinement using data from t = 0 c) and 470 min d); e) Comparison of the experimental XRPD pattern achieved at t = 470 min with simulated patterns; f) Conceptual model of carbon species at 470 min. Reproduced with permission.^[^ ^56^ ^]^ Copyright 2024, Wiley (CC‐BY 4.0).

Aumond et al.^[^ ^56^ ^]^ utilized in situ XRD to study the development of zeolite‐templated carbons in FAU zeolite (**Figure** **9**). By employing SR‐XRD, the researchers monitored in real‐time the formation and development of carbon materials within the zeolite channels. The results indicated that the formation of carbon materials occurred in several stages, including the adsorption of carbon precursors, pyrolysis, and the eventual structural evolution of the carbon. By analyzing the changes in the crystal structure from the diffraction patterns, particularly the shifts and intensity variations of diffraction peaks, the researchers revealed the ordered development of the carbon materials within the FAU zeolite channels, demonstrating that the process was closely linked to the pore structure of the zeolite template.

Polisi et al.^[^ ^57^ ^]^ applied in situ SR‐XRD and Fourier Transform Infrared Spectroscopy (FTIR) to study the adsorption and desorption process of CO_2_ in FAU zeolite nanocrystals in real time. SR‐XRD reveals the subtle changes in the crystal structure and pore channels of FAU zeolite during CO_2_ adsorption and desorption by monitoring the shifts in diffraction peak positions and intensities. FTIR analyzes the variations in the characteristic absorption peaks of CO_2_, particularly the changes in the stretching vibration peaks during adsorption and desorption, to uncover the interaction between CO_2_ molecules and the surface of the zeolite, as well as the adsorption state within the pores. The combined use of both techniques provides comprehensive structural and dynamic information for understanding the behavior of CO_2_ in FAU zeolite.

The above examples collectively emphasize the dynamic evolution of zeolite framework structures during synthesis, ion exchange, and post‐synthetic modifications, which are crucial for understanding the factors that influence zeolite stability, flexibility, and catalytic performance.^[^ ^54^, ^55^, ^56^, ^57^ ^]^ These studies demonstrate the ability of in situ SR‐XRD, combined with other methods, to capture real‐time structural changes and mechanisms. This offers valuable insights into how zeolites respond to various synthesis conditions, such as temperature, pressure, and chemical treatments, providing a comprehensive understanding of the processes driving structural transformations, phase stability, and defect behavior. The findings from these studies are crucial for the rational design and optimization of zeolite‐based catalytic materials and ion‐exchange systems.

## 4 Applications of SR‐XRD in Zeolite Catalysis Research

Zeolites are widely used as catalysts in various chemical reactions, particularly in petrochemical and environmental catalysis.^[^ ^38^, ^58^, ^59^ ^]^ For example, SAPO‐34 zeolite is employed in the methanol‐to‐olefins (MTO) process, which is extensively used for producing olefins like ethylene and propylene, key raw materials for many chemical products.^[^ ^58^ ^]^ With their excellent pore structure and tunability, zeolite catalysts demonstrate outstanding catalytic performance in a variety of reactions. Zeolite catalysts exhibit complex dynamic behavior under working conditions, including framework deformation, pore structure modification, and active site migration or transformation. Understanding these phenomena is critical for unraveling the structure‐function relationships that govern catalytic activity, selectivity, and stability. SR‐XRD provides unique capabilities to monitor such structural evolutions in situ, offering atomic‐ to mesoscale insights into the catalyst behavior under reaction conditions.^[^ ^58^, ^59^, ^60^, ^61^, ^62^, ^63^, ^64^, ^65^, ^66^, ^67^, ^68^, ^69^ ^]^

### 4.1 Structural Evolution of Zeolite Frameworks

#### 4.1.1 Framework Breathing and Lattice Expansion under Catalytic Reactions

During catalytic processes, zeolite frameworks are not static; instead, they often exhibit flexible “breathing” behavior in response to adsorption and reaction intermediates. This expansion or contraction alters the effective pore sizes and diffusion pathways, thereby influencing catalytic performance.^[^ ^58^, ^59^, ^60^ ^]^

**Figure 10:**
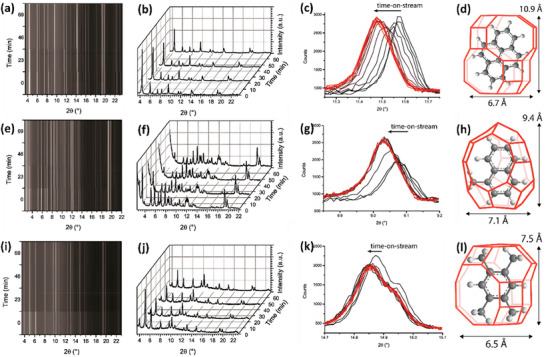
Analysis of zeolite frameworks (CHA, DDR and LEV). (λ=0.709 Å) CHA: a) contour, b) operando XRD patterns, c) 104 peak shift of single XRD peaks and d) lattice expansion; DDR: e) contour, f) operando XRD patterns, g) 211 peak shift of single XRD peaks and h) lattice expansion; LEV: i) contour, j) operando XRD patterns, k) 211 peak shift of single XRD peaks and l) lattice expansion during the conversion of methanol. Reproduced with permission.^[^ ^58^ ^]^ Copyright 2018, American Chemical Society (CC‐BY‐NC‐ND).

Goetze et al.^[^ ^58^ ^]^ used in situ XRD and UV–Vis spectroscopy to investigate the lattice expansion of small‐pore zeolite catalysts during the methanol‐to‐olefins (MTO) process (**Figure** **10**). In situ XRD analysis allowed real‐time monitoring of lattice changes in the zeolite during the reaction, revealing significant lattice expansion of the catalyst. The UV‐Vis spectroscopy results showed a correlation between lattice expansion and changes in the acid sites and catalytic performance of the catalyst. Furthermore, the dynamic structural changes of the catalyst were revealed, including alterations in acidic sites and the evolution of metal species on the catalyst surface. These findings demonstrate that lattice expansion is closely linked to the catalyst's performance, highlighting its crucial role in the MTO process.

Wragg et al.^[^ ^59^ ^]^ employed in situ SR‐XRD to directly observe the behavior of SAPO‐18 and SAPO‐34 catalysts under real working conditions for MTO reactions. The results revealed that both catalysts exhibited significant changes in their crystalline structures during the reaction. SAPO‐34 showed a more stable crystalline structure with negligible lattice changes, while SAPO‐18 showed significant lattice expansion and displayed more rapid structural transformations, particularly under methanol‐rich conditions. This indicates that SAPO‐34 possesses better structural stability during the MTO process, resulting in a longer catalytic lifetime compared to SAPO‐18. The SR‐XRD technique revealed the changes in catalyst performance and structural stability during the MTO process, offering valuable insights into the structure‐performance relationship of small‐pore zeolite catalysts and guiding catalyst design optimization.

**Figure 11:**
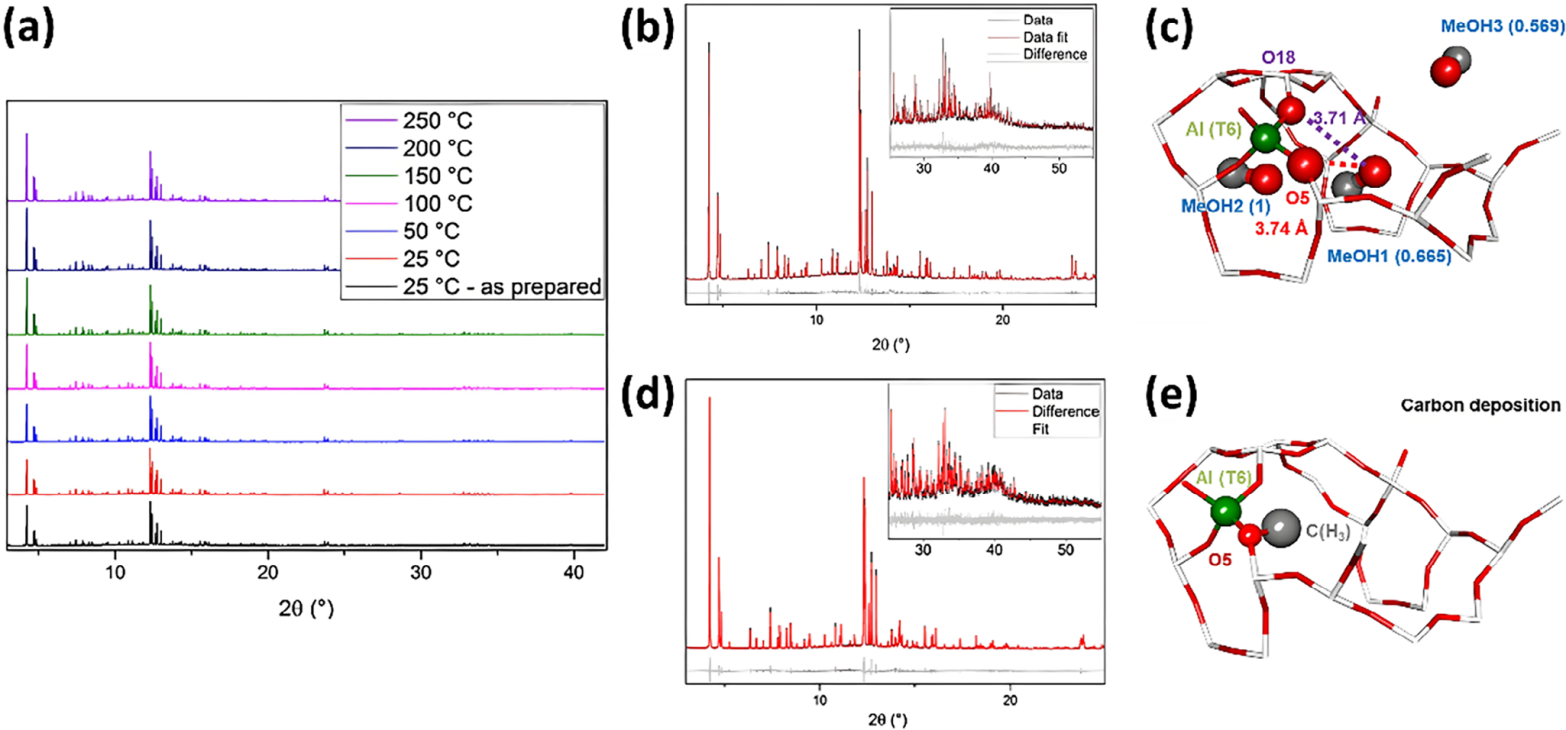
Analysis of H‐ZSM‐5 zeolite in monitoring the methanol conversion process. a) SR‐XRD patterns (25–250 °C); b) SR‐XRD patterns and Rietveld refinement of H‐ZSM‐5 sample at 25 °C; c) Corresponding Rietveld derived crystal structure; d) SR‐XRD patterns and Rietveld refinement of H‐ZSM‐5 sample for 24 h at 200 °C; e) Corresponding Rietveld derived crystal structure showing one asymmetric unit of a unit cell. Reproduced with permission.^[^ ^60^ ^]^ Copyright 2018, Elsevier.

Lo et al.^[^ ^60^ ^]^ employed SR‐XRD and Mass Spectrometry (MS) to monitor the framework changes of H‐ZSM‐5 during methanol conversion to hydrocarbons (MTH) (**Figure** **11**). Using SR‐XRD, the researchers tracked the subtle changes in the crystalline structure of the H‐ZSM‐5 catalyst during the reaction, particularly under high‐temperature conditions. The MS analysis provided detailed information on the intermediates and products generated during the process. Combining the results from both techniques, the results show that, at 200 °C, in the early stages of methanol conversion, methanol primarily undergoes an associative mechanism, with methanol molecules adsorbed on the catalyst surface reacting to form intermediates. As the reaction progresses, minor structural changes in the H‐ZSM‐5 framework are observed. A small amount of methoxy intermediate decomposes at Brønsted acid sites, leading to methoxy accumulation in the later stages of the reaction. These structural changes are closely linked to the catalyst's activity and stability, indicating that the stability of the framework is crucial for long‐term catalyst performance.

**Figure 12:**
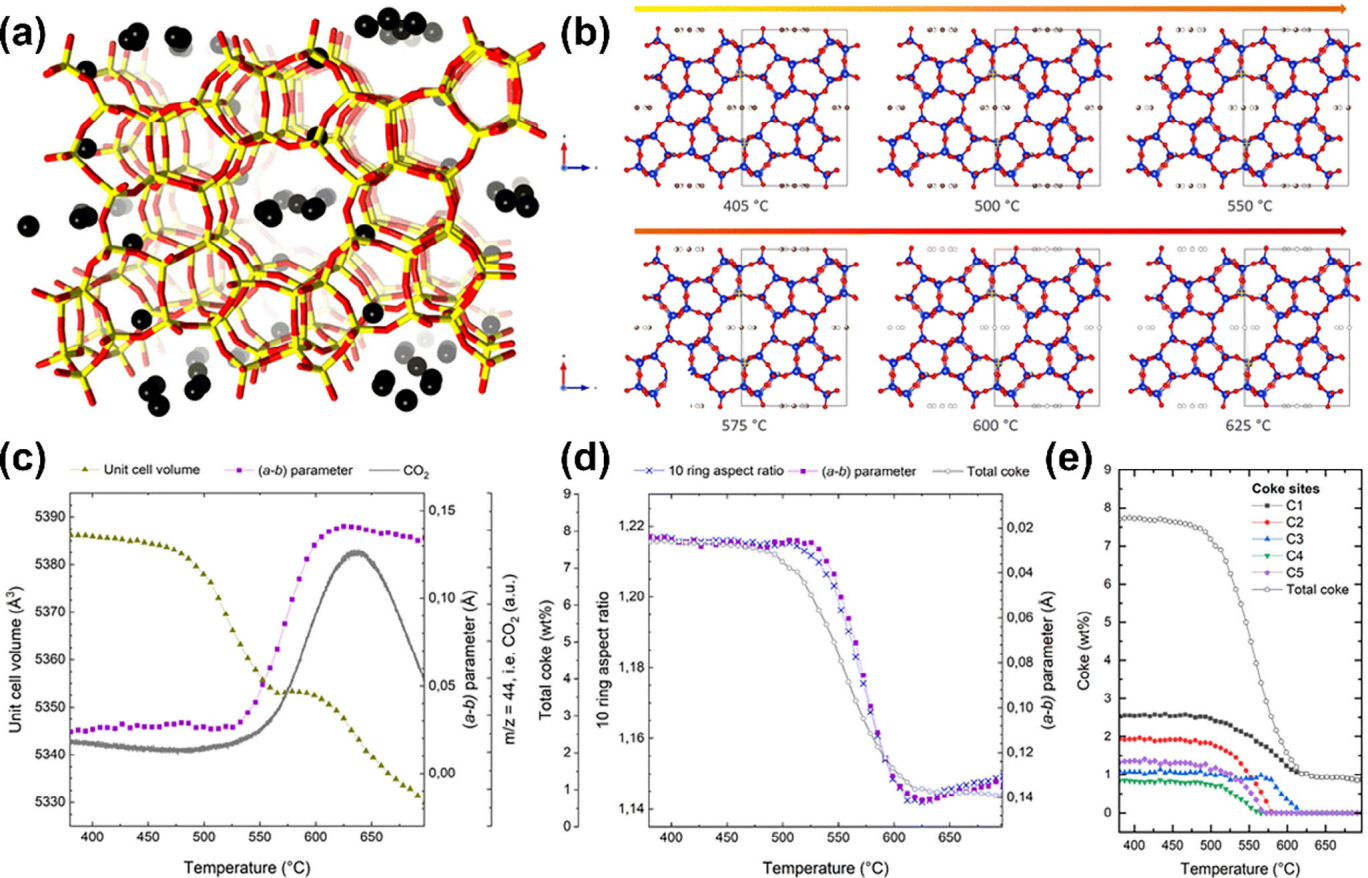
Structural evolution and coke dynamics during the regeneration of H‐ZSM‐5 catalysts. a) Unit cell structure of H‐ZSM‐5 viewed along the b‐axis. b) Graphical representation of the framework rearrangement extracted from calculated CIF files. c) Evolution of CO_2_ signal, (a‐b) lattice parameter, and unit cell volume during catalyst regeneration. d) Evolution of total coke content, (a,b) lattice parameter, and 10‐ring aspect ratio throughout the regeneration process. (e) Temperature‐dependent evolution of total coke and individual coke occupancies within the H‐ZSM‐5 framework. (λ=0.7743 Å) Reproduced with permission.^[^ ^61^ ^]^ Copyright 2022, Royal Society of Chemistry (CC‐BY).

In the reaction of methanol conversion to gasoline (MTG), SR‐XRD and crystallographic imaging were employed by Kalantzopoulos et al.^[^ ^61^ ^]^ to monitor the real‐time regeneration of a working zeolite, particularly focusing on the removal of coke from the MFI framework. (**Figure** **12**). Using operando XRD, the researchers observed that during the regeneration process, coke was gradually removed from the MFI framework, accompanied by subtle changes in the crystal structure. Crystallographic imaging further revealed that the catalyst structure remained stable during regeneration, and the removal of coke facilitated the restoration of catalytic activity. The findings offer important insights into the dynamic process of coke deposition and removal, as well as the regeneration mechanism, contributing to a better understanding of catalyst deactivation and longevity in MTG reactions.

These findings demonstrate that SR‐XRD in catalytic reactions, offering real‐time structural data that reveals the dynamic changes in catalyst frameworks throughout the reaction process. Additionally, SR‐XRD combined with other characterization techniques aid in gaining a deeper understanding of the key mechanisms in catalytic reactions, providing essential theoretical support for catalyst design and optimization.^[^ ^58^, ^59^, ^60^, ^61^ ^]^

#### 4.1.2 Phase Transitions and Framework Changes under Certain Conditions

Zeolite frameworks are prone to phase transitions under thermal or chemical stress, especially when exposed to high temperatures, pressures, or harsh chemicals. These conditions can cause the framework to shift between polymorphs or transform into a less stable structure. In extreme cases, the framework may collapse irreversibly, losing its microporosity and catalytic activity. These structural changes severely affect catalytic performance, often reducing efficiency and lifespan. Monitoring these transformations is essential for optimizing catalytic processes and improving the stability of zeolite catalysts under industrial conditions.^[^ ^62^, ^63^, ^64^, ^65^ ^]^

**Figure 13:**
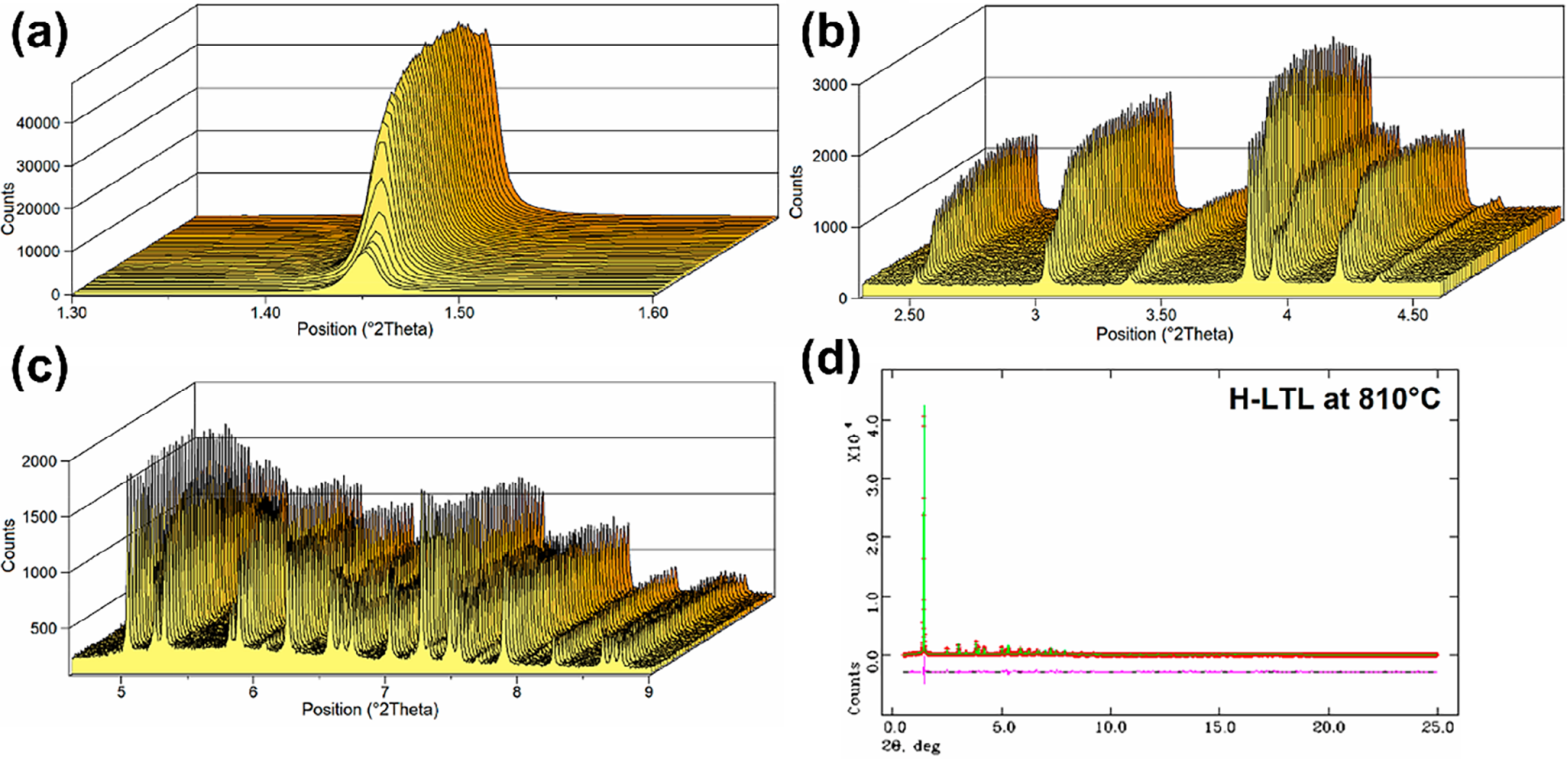
SR‐XRD data from RT to 810 °C in three magnified angular ranges: a) 1.3‐1.6°(2θ), b) 2.35‐4.75°(2θ), and c) 4.75‐9.0°(2θ). d) Rietveld refinement plot for the zeolite H‐LTL collected at 810 °C. (λ=0.40003 Å) Reprinted with permission from.^[^ ^62^ ^]^ Copyright 2023, American Chemical Society.

Precisvalle et al.^[^ ^62^ ^]^ used time‐resolved SR‐XRD to study the temperature‐induced structural changes in acidic L‐zeolite (**Figure** **13**). The results showed that during heating, significant structural changes occurred, especially at high temperatures, where the lattice structure of the L‐zeolite exhibited noticeable expansion and contraction. By monitoring the XRD patterns at different temperatures in real‐time, the researchers found that the framework of acidic L‐zeolite underwent significant phase transitions at high temperatures, which has important implications for its catalytic performance.

**Figure 14:**
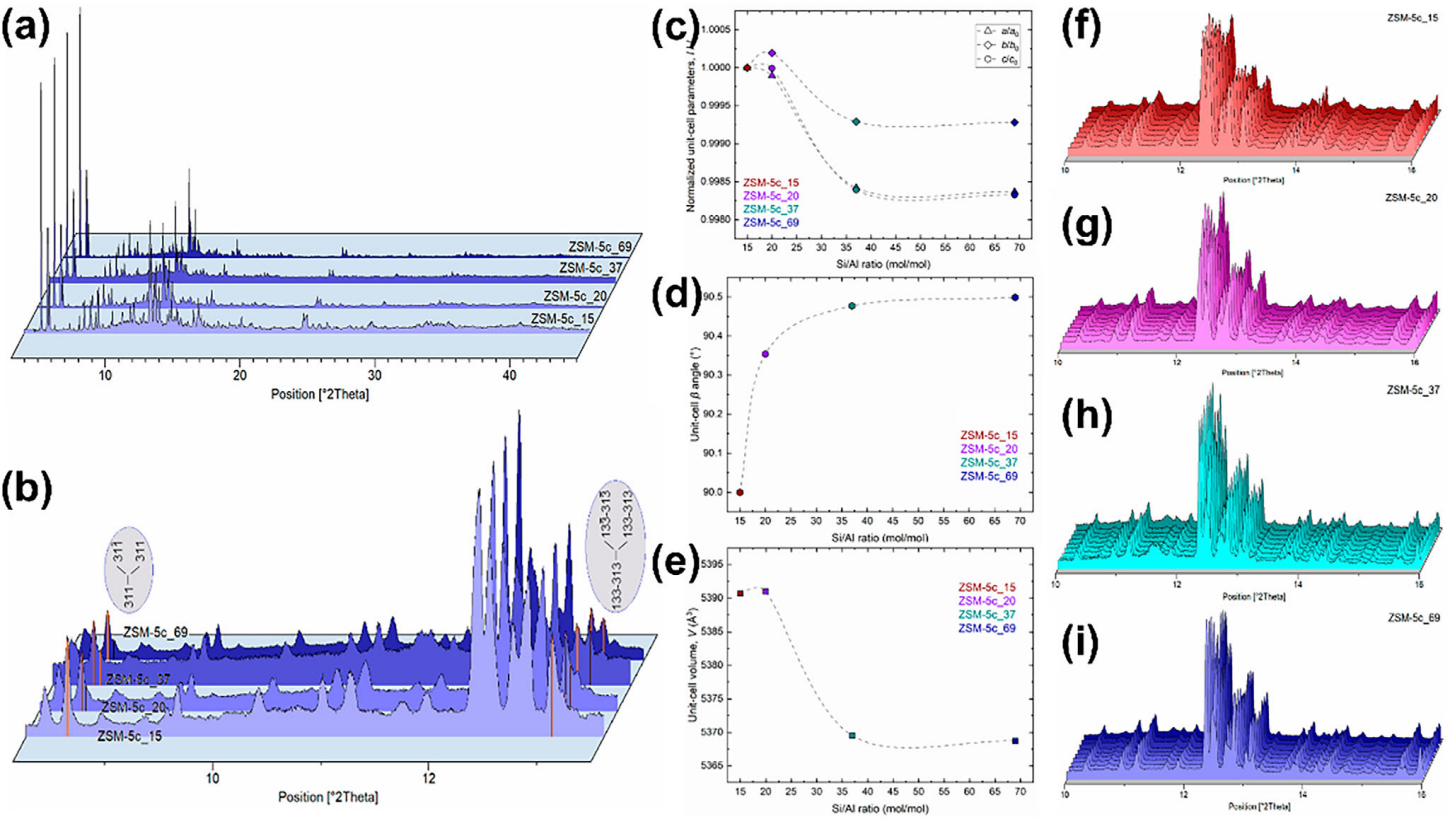
XRD patterns of all ZSM‐5 samples in the entire angle range a) and in the angle range 8.0‐14.0 b); Lattice parameters c), β angle d) and unit‐cell volume e) of the four investigated ZSM 5c zeolite samples as a function of the Si/Al ratio at room temperature; X‐ray powder diffraction patterns collected at high temperature (details of high angles) for samples ZSM‐5c_15 f), ZSM‐5c_20 g), ZSM‐5c_37 h) and ZSM‐5c_69 i). (λ=0.827 Å) Reproduced with permission.^[^ ^63^ ^]^ Copyright 2023, MDPI (CC‐BY).

Precisvalle et al.^[^ ^63^ ^]^ also investigated the phase transition of protonated ZSM‐5 with varying Si/Al ratios using in situ SR‐XRD (**Figure** **14**). They monitored the monoclinic‐to‐orthorhombic phase transformation as a function of temperature, highlighting that the framework symmetry changes were sensitive to aluminum content. By analyzing real‐time XRD data, the study revealed the impact of the Si/Al ratio on the structural stability and phase transition behavior of the zeolite. Specifically, ZSM‐5 with a lower Si/Al ratio was more prone to phase transition and tended to transform into the orthorhombic phase at higher temperatures. Such phase transitions influence the accessibility of active sites and the diffusion of reactants and products, thereby affecting catalytic performance. These findings provide valuable structural insights for understanding the thermal stability of ZSM‐5 and optimizing its catalytic performance.

Mancinelli et al.^[^ ^64^ ^]^ used in situ SR‐XRD to track the thermal stability of template‐containing ZSM‐5. By tracking the crystal structure changes of the zeolite at various temperatures in real‐time, the researchers observed significant structural alterations in templated ZSM‐5 at high temperatures, primarily manifesting as framework degradation and lattice contraction. The analysis indicated that the presence of the template plays a crucial role in the thermal stability of ZSM‐5. Templated ZSM‐5 exhibited higher structural stability at lower temperatures, but at higher temperatures, the removal of the template led to significant structural damage. By analyzing the XRD data, the study revealed how the structural features introduced during the templating process affect the thermal stability of the zeolite.

**Figure 15:**
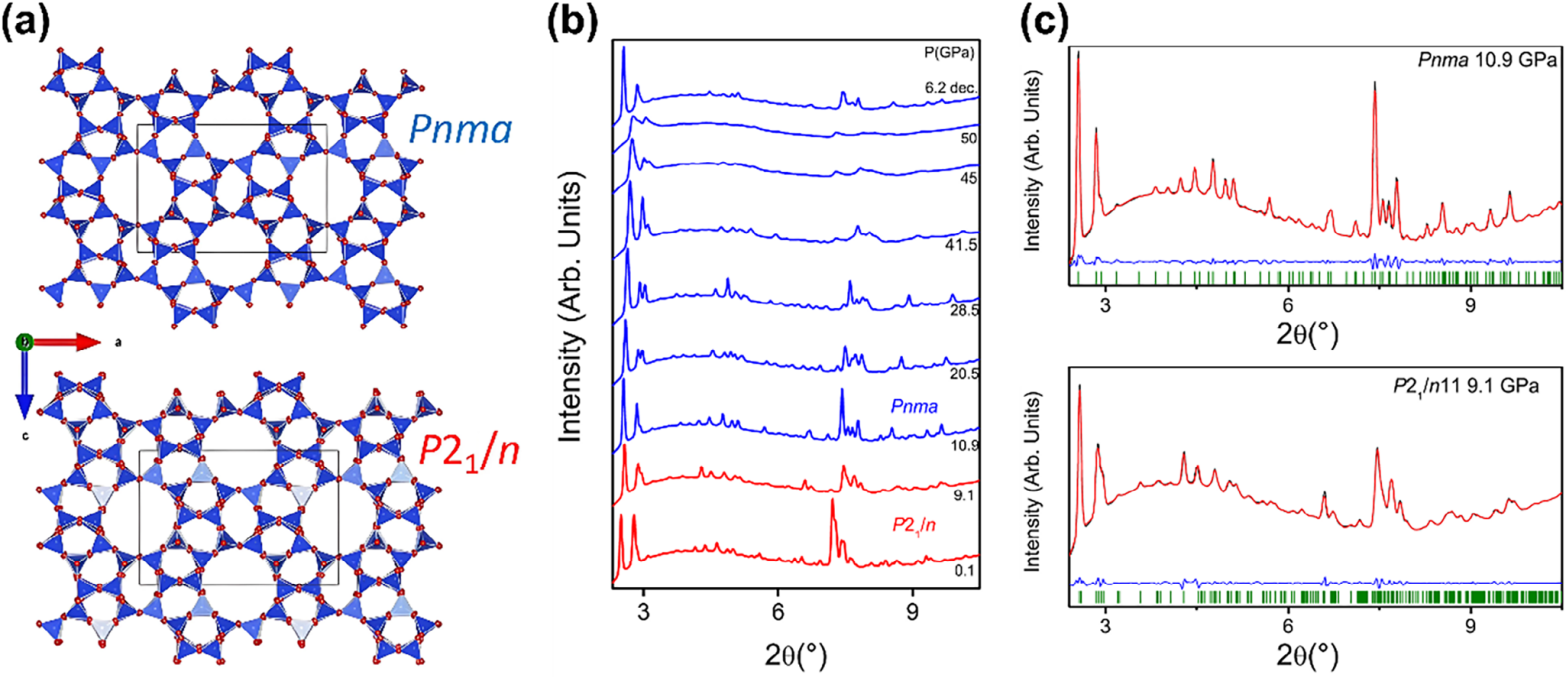
a) Structures of monoclinic P2_1_/n, space group 14 (below), and orthorhombic Pnma, S.G. 62 MFI (above); b) X‐ray diffraction patterns of MFI in He as a function of pressure; (λ=0.4828 Å) c) Experimental (black), calculated (red), and difference (blue) profiles for the Le Bail fits for the P2_1_/n structure of MFI/He at 9.1 GPa (below) and for the Pnma structure of MFI/He at 10.9 GPa (above). Reprinted with permission from.^[^ ^65^ ^]^ Copyright 2021, American Chemical Society.

SR‐XRD was used by Santoro et al.^[^ ^65^ ^]^ to monitor the structural changes of the siliceous zeolite Mobil‐Five under high‐pressure helium conditions (**Figure** **15**). The XRD data revealed a phase transition from monoclinic to orthorhombic symmetry, accompanied by a positive volume change, as a result of the high‐pressure insertion of dense fluid helium, which caused a rearrangement of the internal structure and expansion of the framework. This technique provided precise information about the atomic arrangement and the strain distribution within the zeolite framework, offering a detailed understanding of the structural evolution during the phase transition induced by fluid pressure.

Understanding phase behavior under reaction conditions is thus vital for predicting catalyst stability and tailoring zeolite compositions for enhanced resilience. Together, these investigations highlight the dynamic nature of zeolite frameworks under catalytic conditions.^[^ ^62^, ^63^, ^64^, ^65^ ^]^ Monitoring framework evolution using SR‐XRD not only elucidates reaction mechanisms but also informs strategies for improving catalyst design, such as optimizing framework flexibility, mitigating phase instability, and enhancing thermal resilience.

### 4.2 Tracking and Evolution of Catalytic Active Sites

#### 4.2.1 Metal Cation Migration and Sintering

Active metal cations in zeolite pores can move, sinter, or agglomerate during catalytic reactions, affecting performance. These changes occur when metal ions shift within the zeolite framework, often due to high temperature, pressure, or reactants. This alters the distribution of active sites, impacting catalytic activity and selectivity.^[^ ^66^, ^67^, ^68^ ^]^ Metal cations may also form larger particles, reducing catalytic efficiency. Sintering decreases surface area and increases deactivation risk. Migration can destabilize the zeolite structure, further lowering the catalyst's effectiveness and lifespan.

**Figure 16:**
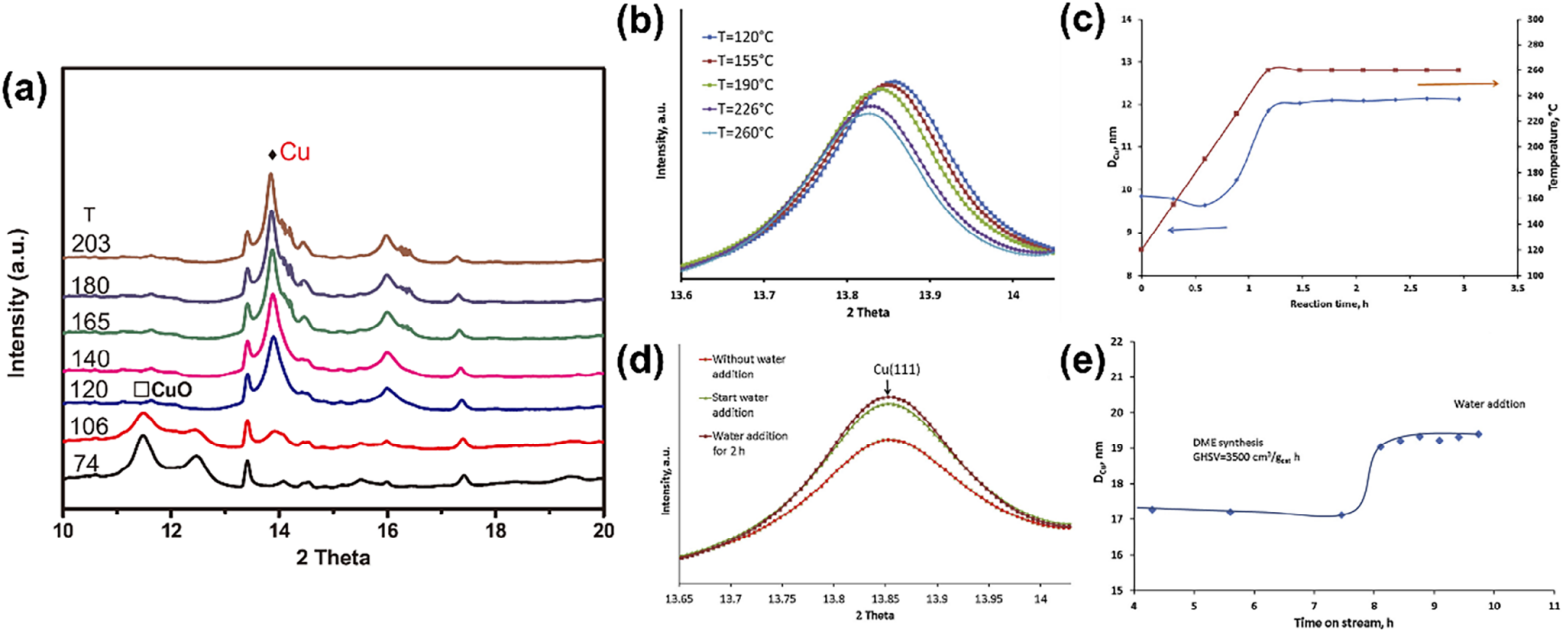
a) In situ XRD patterns measured during reduction of the Cu‐Zn‐Al (CZA)/ZSM‐5 catalyst. Evolution of the Cu (111) XRD peak b) and copper metal nanoparticle size calculated using the Scherrer method c) at high gas space velocity. Evolution of the Cu (111) peak d) and copper metal nanoparticle size calculated using the Scherrer method (e) during direct DME synthesis at low gas space velocity and with the addition of water. Reproduced with permission.^[^ ^66^ ^]^ Copyright 2020, Elsevier.

Khodakov et al.^[^ ^66^ ^]^ applied SR‐XRD and XAFS to study copper sintering during direct dimethyl ether synthesis over Cu‐zeolite catalysts (**Figure** **16**). It analyzed the changes in the catalyst's crystal structure via XRD (to observe whether there were diffraction peak broadening or intensity changes caused by the growth of metal particles) and examined the coordination environment, valence state, and dispersion state of copper species through XAS. They found that metallic copper aggregation reduced the dispersion of active sites, significantly decreasing catalytic activity and durability. These findings underscore the importance of stabilizing metal cations against migration and sintering under reaction conditions.

Lee et al.^[^ ^67^ ^]^ provided new insights into cation relocations within the pores of zeolite Rho by using in situ SR‐XRD and neutron powder diffraction studies on Pb‐ and Cd‐exchanged Rho. SR‐XRD directly trac k the precise positions and temperature‐dependent occupancy changes of heavy metal cations (Pb^2^⁺/Cd^2^⁺) within the pores of zeolite Rho, revealing their migration path from eight‐membered rings toward six‐membered rings upon heating. Simultaneously, neutron powder diffraction was used to precisely resolve the zeolite framework structure and locate deuterated water molecules and deuterons interacting with the framework, clarifying the changes in the cations' hydration shells and their interactions with the framework during migration. The combination of both techniques comprehensively unveiled the atomistic mechanism of the dynamic cation relocation.

Norby et al.^[^ ^68^ ^]^ investigated cation migration in zeolite Cs (Na)‐Y during dehydration using in situ XRD and MAS NMR techniques. It real‐time tracked changes in the zeolite's crystal structure via in situ SR‐XRD, observing the positional shift of diffraction peaks corresponding to cations to determine changes in the cations' spatial positions. Meanwhile, it used MAS NMR to analyze the local chemical environment of the zeolite framework and cations, revealing adjustments in the interaction between cations and framework oxygen through changes in chemical shifts. The results show that Cs and Na cations migrate during dehydration, and the SR‐XRD data reveal significant changes in the zeolite's crystal structure at different temperatures. Particularly, during high‐temperature dehydration, the Cs cations migrate from their initial positions to different pores in the zeolite, causing partial structural changes.

The above indicates that SR‐XRD can detect the changes in metal cations within zeolite pores, such as migration, sintering, and agglomeration, during catalytic reactions.^[^ ^66^, ^67^, ^68^ ^]^ By providing real‐time monitoring of the metal particle evolution and structural shifts in the zeolite framework, SR‐XRD helps track the effects of high temperature, pressure, and reactants on catalyst performance, aiding in the design of more stable and efficient catalysts.

#### 4.2.2 Coke Formation and Its Impact on Active Sites

In the field of zeolite catalysis, carbonaceous deposits, commonly referred to as “coke,” are formed during hydrocarbon processing reactions. These deposits can accumulate on the catalyst surface and within the pores of the zeolite, leading to pore blockage and deactivation of the catalytic active sites. To understand the impact of coke formation on catalyst performance, SR‐XRD has been widely used for detailed structural characterization. SR‐XRD allows for high‐resolution analysis of the zeolite's crystal structure, providing valuable insights into unit cell expansion or contraction due to coke accumulation.^[^ ^69^, ^70^, ^71^, ^72^ ^]^

**Figure 17:**
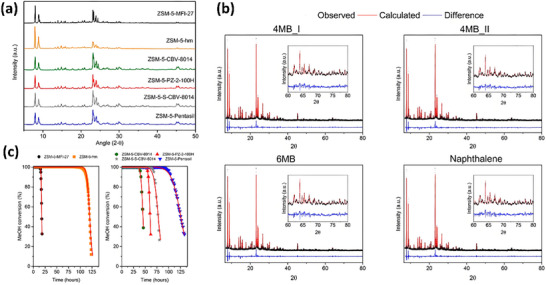
a) XRD patterns of the fresh ZSM‐5 catalysts; (λ=0.7743 Å) b) Rietveld fit for ZSM‐5 coked with 4MB_I, 4MB_II, 6 MB, and naphthalene; c) Conversion of methanol at increasing time on stream over the various ZSM‐5 samples. Reprinted with permission from.^[^ ^71^ ^]^ Copyright 2017, American Chemical Society.

Zhou et al.^[^ ^69^ ^]^ employed a series of characterization techniques, including XRD and Gas Chromatography‐Mass Spectrometry (GC‐MS), to investigate the process of coke formation and its impact on β‐zeolite performance during catalytic cracking. Among these, XRD was primarily used to analyze structural changes in the catalyst, revealing crystal structure degradation during coke accumulation. GC‐MS helped analyze the chemical composition of the coke, providing qualitative and quantitative information on the organic compounds formed during the coke generation process. The results reveal that coke accumulation leads to pore blockage and structural changes in the β‐zeolite framework, which ultimately causes a significant reduction in catalytic activity. Additionally, the transformation of coke during the cracking process was observed, showing that coke reorganization and interaction with the catalyst surface contribute to catalyst deactivation. The study also discusses the effect of reaction conditions on coke formation and its influence on reaction selectivity. Rojo‐Gama et al.^[^ ^70^, ^71^ ^]^ studied the deactivation process of the H‐ZSM‐5 zeolite catalyst during the MTG reaction using in situ time‐ and space‐resolved XRD (**Figure** **17**). The study reveals that during the MTG reaction, the zeolite framework undergoes significant changes, including pore blocking and structural collapse, which lead to the gradual deactivation of the catalyst. The results show that these structural changes are closely linked to the accumulation of carbonaceous deposits and the formation of coke, which obstruct the active sites and reduce the catalyst's effectiveness. By using time‐ and space‐resolved XRD, the evolution of the catalyst's structure during the reaction was tracked, providing insights into the mechanisms of catalyst deactivation.

Zokaie et al.^[^ ^72^ ^]^ employed SR‐XRD and DFT to investigate the unit cell expansion of a SAPO‐34 catalyst upon coke formation during the MTO reaction. In experiments, XRD was used to determine diffraction data of SAPO‐34 with different coke amounts, calculate changes in unit cell parameters (e.g., unit cell volume), and track their evolution with increasing coke content. Computationally, DFT simulated the adsorption and interaction of coke species (such as polycyclic aromatic coke precursors) in SAPO‐34 pores, analyzing the effect of coke on the framework structure from a theoretical perspective. The results indicated that the unit cell of SAPO‐34 expanded as coke deposited on the catalyst, suggesting structural changes due to the accumulation of carbonaceous species. This expansion was correlated with the degree of coke formation and the catalyst's deactivation.

Based on the examples above, SR‐XRD plays a key role in studying coke formation, particularly in the structural analysis of catalyst deactivation. It helps establish a direct link between coke accumulation and catalyst deactivation, providing deeper insights into the mechanisms underlying catalyst deactivation.^[^ ^69^, ^70^, ^71^, ^72^ ^]^

#### 4.2.3 Acid Site Distribution and Interaction with Adsorbates

The location and configuration of Brønsted acid sites in zeolite, which are primarily influenced by the distribution of aluminum within the framework, play critical roles in catalytic processes. The precise characterization of these acid sites is essential for understanding the underlying mechanisms of catalysis. SR‐XRD technique has been proven to be invaluable in providing detailed insights into the structural properties of these acid sites.^[^ ^73^, ^74^, ^75^, ^76^ ^]^ By utilizing SR‐XRD, researchers can effectively map the distribution of aluminum atoms and their impact on the framework's overall architecture, thus allowing for a more accurate determination of the Brønsted acid site density and configuration.^[^ ^73^, ^74^, ^75^, ^76^ ^]^

**Figure 18:**
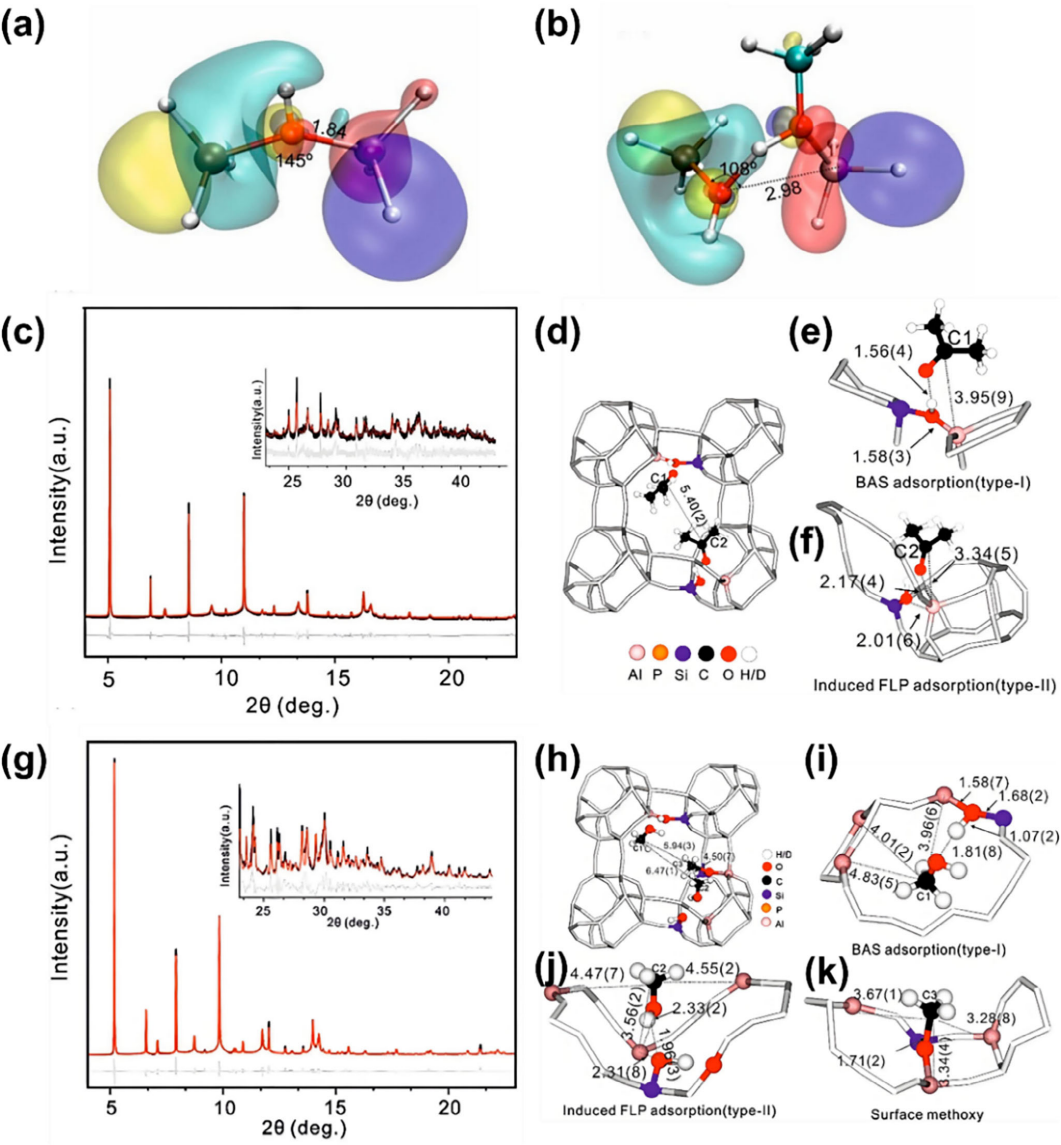
HOMO and LUMO of zeolite clusters extracted from optimized periodic H‐SAPO‐34 zeolite by replacing terminal Al and Si by H before a) and after adsorption of methanol b). c) SXRD data measured at 298 K of acetone in H‐SAPO‐34. Crystallographic model d), acetone adsorbed on BAS, type‐I e) and the framework Al site; type‐II (symmetry in adsorption sites is disregarded for clarity f). g) SXRD data measured at 298 K of methanol in H‐SAPO‐34. Crystallographic model h), methanol adsorbed on BAS, type I i), and the framework Al site, type II j), and the formed surface methoxy species k). Reproduced with permission.^[^ ^74^ ^]^ Copyright 2021, American Chemical Society.

Li et al.^[^ ^73^ ^]^ used SR‐XRD as the core experimental method, combined with DFT calculations, to investigate the spatial distribution of isolated aluminum atoms vs aluminum pairs in H‐ZSM‐5. SR‐XRD collected atomic‐level diffraction data of H‐ZSM‐5. By analyzing the fine splitting and intensity changes of characteristic diffraction peaks corresponding to framework atoms (Si, Al, O) in the XRD pattern, it accurately located the specific occupancy of Al single atoms at the framework T‐sites (tetrahedral coordination sites) of the zeolite (e.g., T1, T2 sites) and further distinguished the lattice spacing and relative positions of Al pair sites (e.g., adjacent T‐sites or spaced T‐sites). Their findings showed that the proximity of acid sites influences adsorption strength and reaction pathways, with paired sites often facilitating bifunctional catalytic mechanisms. Moreover, adsorbate interactions were found to induce local framework distortions, modulating acid strength dynamically during catalysis. Li et al.^[^ ^74^ ^]^ also studied the effect of adsorbates on the induction of active sites in zeolite SAPO employing SR‐XRD (**Figure** **18**). With SR‐XRD, the researchers were able to monitor the structural changes of the zeotype framework in real time during the adsorption process, revealing how adsorbates influence the local structure of the zeolite and induce the formation of new active sites. The study revealed that Brønsted acid sites in zeolite SAPO are induced to form blocked Lewis acid‐base pairs structures during the reaction, significantly enhancing catalytic activity. It was also found that this structure has a lower activation barrier in methanol dehydration, further improving catalytic performance.

Sen et al.^[^ ^75^ ^]^ used SR‐XRD to examine the high‐temperature thermal expansion behavior of H[Al]ZSM‐5 zeolites and the role of Brønsted acid sites in this process. SR‐XRD allowed the researchers to precisely monitor the changes in the crystal structure of the zeolite under high‐temperature conditions, revealing the influence of acid sites on the thermal expansion behavior. The results show that the presence of Brønsted sites significantly influences the thermal expansion of the zeolite structure. SR‐XRD data reveals that the zeolite undergoes a reversible expansion upon heating, with changes in the unit cell dimensions. The expansion is more pronounced in the presence of Brønsted acid sites, suggesting that these sites play a key role in stabilizing the framework during thermal treatment and affecting the overall thermal behavior of the material.

Chen et al.^[^ ^76^ ^]^ employed SR‐XRD and FTIR to investigate the catalytic behavior of ketene intermediates in zeolite materials and explore the effect of confinement on reaction pathways. SR‐XRD was used to monitor the structural changes of the zeolite under different reaction conditions, revealing the confinement effect of the zeolite channels on the ketene intermediates and the resulting structural deformations. FTIR was employed to detect the adsorption modes and reactivity of ketene intermediates on the zeolite surface, further validating the results obtained from SR‐XRD. The results reveal that confinement within the catalyst pores induces significant changes in the arrangement of ketene intermediates, which alters their stability and reactivity.

Understanding acid site dynamics under reaction conditions is essential for tailoring zeolite catalysts with optimized activity and selectivity. Overall, SR‐XRD studies reveal that catalytic active sites are highly dynamic entities, evolving in response to reaction environments. Monitoring their migration, aggregation, and interactions with adsorbates provides mechanistic insights critical for catalyst optimization and rational design.^[^ ^73^, ^74^, ^75^, ^76^ ^]^

## 5 Current Challenges and Future Perspectives for SR‐XRD in Zeolite Research

Despite the remarkable advances enabled by SR‐XRD in zeolite research, several technological limitations persist, restricting its broader application in dynamic, multiscale, and complex catalytic systems.^[^ ^77^, ^78^, ^79^, ^80^, ^81^, ^82^, ^83^ ^]^ Concurrently, emerging developments in multimodal characterization and artificial intelligence offer promising avenues to overcome these challenges.^[^ ^82^, ^84^, ^85^, ^86^, ^87^, ^88^, ^89^, ^90^, ^91^, ^92^, ^93^, ^94^, ^95^, ^96^, ^97^, ^98^, ^99^, ^100^, ^101^ ^]^ This section systematically discusses the current technical barriers and outlines potential directions for future advancements.

### 5.1 Current Technical Challenges

#### 5.1.1 Limitations in Characterization under Extreme Conditions

In situ SR‐XRD experiments under extreme conditions‐such as high temperatures (>800 °C) and high pressures (>100 bar)‐often suffer from degraded signal‐to‐noise ratios. Thermal expansion, phase transitions, and chemical transformations induce diffraction signal weakening and increased background noise, complicating data acquisition and interpretation.^[^ ^77^, ^78^, ^79^ ^]^ Current instrumentation faces difficulties in capturing subtle structural changes under such demanding environments, impeding real‐time monitoring of zeolite frameworks during harsh catalytic processes.

#### 5.1.2 Challenges in Multiscale Structural Correlation

Zeolites often exhibit hierarchical structures spanning from atomic to micrometer scales, especially in aggregated catalyst forms. Capturing and correlating structural features across these scales remains a technical hurdle. Laboratory XRD‐based techniques primarily resolve average crystallographic information, while nanoscale heterogeneities and mesoscale aggregation effects often elude detection.^[^ ^73^, ^80^, ^81^ ^]^ The lack of integrated multiscale characterization methods limits comprehensive understanding of structure‐property relationships in complex zeolitic materials.

#### 5.1.3 Complexity of High‐Dimensional Diffraction Data Analysis

With advances in time‐resolved and energy‐resolved SR‐XRD, SR‐XRD high‐dimensional datasets are obtained through multi‐parameter synchronic collection, primarily consisting of three key dimensions, with diffraction information further layered across three independent dimensions. The time‐resolved dimension captures diffraction patterns at millisecond or microsecond intervals, such as taking one image every 10 ms to monitor zeolite crystallization, forming a 3D dataset of “Time×Diffraction Angle×Diffraction Intensity.” For a 1 h experiment, this can generate 36 000 images, totaling ≈100GB of data. The energy‐resolved dimension scans different X‐ray energies (e.g., 0.1–100 keV), collecting diffraction signals at each energy level, forming a 3D dataset of “Energy×Diffraction Angle×Diffraction Intensity,” commonly used to analyze element distributions (e.g., aluminum). The spatially resolved dimension performs 2D scans of specific catalyst particle areas (e.g., a 50×50 µm region, scanning every 1 µm), producing a 3D dataset of “Spatial Position×Diffraction Angle×Diffraction Intensity.” Combining the three dimensions‐time, energy, and space‐expands the dataset to “Time×Energy×Spatial Position×Diffraction Angle×Diffraction Intensity,” increasing the data size to the TB level. For example, high‐dimensional data from a 1 h experiment can reach ≈1 TB. Laboratory analysis approaches struggle to efficiently process such datasets, leading to information bottlenecks and under‐utilization of valuable structural insights. Particularly during dynamic catalytic processes, the inability to extract critical features rapidly hinders real‐time understanding and catalyst optimization.^[^ ^61^, ^82^, ^83^ ^]^

### 5.2 Future Perspectives

#### 5.2.1 Technological Innovations

##### Integration with X‐Ray Free Electron Lasers (XFELs)

XFELs integration with zeolite research provides new opportunities for studying ultrafast structural dynamics. The comparison between XFELs and Synchrotron Radiation (SR) is shown in **Table** **3**. XFELs can generate femtosecond X‐ray pulses with extremely high brilliance, enabling real‐time observation of bond formation, lattice distortions, and transient intermediates during catalytic reactions.^[^ ^84^, ^85^, ^86^ ^]^ These observations are crucial for understanding the fundamental mechanisms of catalytic processes, as they occur on extremely short timescales and are difficult to capture with conventional X‐ray techniques. The use of XFELs not only accelerates our study of complex materials like zeolites but also offers new possibilities for developing more efficient catalytic systems.

**Table 3:** Comparison of XFELs and SR‐XRD.

Techniques	XFELs	Synchrotron radiation
Peak brightness	Extremely high	Higher, but much lower than XFELs
Pulse length	Femtosecond (fs) to attosecond (as) scale	Tens of picoseconds (ps), much longer than XFELs
Energy range	0.1–25 keV	0.1–100 keV
Equipment cost and scale	Extremely high, with a massive device	Higher, smaller in scale than XFELs
Operational efficiency	Low repetition rate (usually 10–100 Hz), limited operating time	High repetition rate (up to MHz level), strong stability, and long startup time

##### Development of High‐Speed, High‐Sensitivity Detectors

The development of detector technologies, especially hybrid pixel detectors and event‐driven detectors, greatly improves our ability to capture fast structural changes.^[^ ^80^, ^81^, ^82^ ^]^ These advanced detectors offer higher temporal resolution, allowing for precise detection of ultrafast processes. They also feature a wider dynamic range, enabling accurate measurement of signals from weak to strong intensities within a single experiment. Additionally, improved signal‐to‐noise ratios provide clearer, more reliable data, even in noisy environments. These advancements are essential for monitoring rapid structural changes under operando in situ conditions, providing real‐time insights into materials' behaviors during reactions or processes. These breakthroughs are pushing the limits of current scientific understanding and opening new opportunities for exploring dynamic phenomena in fields such as catalysis, materials science, and chemistry.

##### Advancement in In Situ Multimodal Platforms

In the field of zeolites and other porous materials, constructing an in situ multimodal coupling platform by combining SR‐XRD with pair distribution function (PDF), pair distribution function computed tomography (PDF‐CT), as well as XAS, SAXS, IR spectroscopy, and MS is crucial for obtaining a comprehensive understanding of catalytic processes.^[^ ^42^, ^43^, ^44^, ^45^, ^46^, ^47^, ^48^, ^49^, ^50^, ^51^, ^52^, ^53^, ^54^, ^55^, ^56^, ^57^, ^58^, ^59^, ^60^, ^61^, ^62^, ^63^, ^64^, ^65^, ^66^, ^67^, ^68^, ^69^, ^70^, ^71^, ^72^, ^73^, ^74^, ^75^, ^76^ ^]^ SR‐XRD excels at providing long‐range crystal structure information but struggles to capture local short‐range/medium‐range structural changes. PDF compensates for this limitation, as it can reveal atomic short‐range order (e.g., the structure around zeolite acid sites, the morphology of hydrocarbon pools in pores) and even identify the characteristics of low‐crystallinity regions. PDF‐CT further enables 3D spatial resolution, presenting the structural heterogeneity of different catalyst regions and breaking the limitation of average structure analysis. In addition, XAS can offer insights into the local electronic environment around metal centers, SAXS can reveal information on nanoscale structural changes, IR spectroscopy is valuable for studying adsorbed species and bonding interactions, and MS provides crucial data on product distribution and reaction mechanisms. Together, the synergy of these techniques covers multi‐scale analysis from electronic structure and local coordination to long‐range frameworks, offering key support for the design and performance optimization of catalytic materials.

##### Miniaturized and Flexible In Situ Reaction Cells

The development of highly adaptable, compact in situ reactors designed for extreme conditions such as high temperature, high pressure, and reactive atmospheres is crucial for advancing SR‐XRD studies.^[^ ^90^, ^91^, ^92^ ^]^ These miniaturized reactors are essential for simulating industrial environments more accurately, providing a clearer understanding of catalyst behavior under real‐world operating conditions. With their flexibility, these reactors allow for real‐time monitoring of reaction dynamics, enabling researchers to observe structural changes in catalysts during actual catalytic processes. This advancement is key to bridging the gap between laboratory research and industrial applications, as it allows for more effective translation of findings into practical applications, particularly in areas such as energy production, petrochemical processing, and environmental protection.

#### 5.2.2 Computational and AI‐Driven Advances

##### Machine Learning for Diffraction Pattern Analysis

Deep learning models, particularly convolutional neural networks (CNNs), are transforming the analysis of synchrotron radiation diffraction data by automating critical tasks such as phase identification, peak deconvolution, and anomaly detection in complex datasets.^[^ ^82^, ^93^, ^94^ ^]^ These models excel in handling the high‐dimensional and noisy data typical of synchrotron experiments, significantly accelerating the process of data interpretation. By learning from vast datasets generated in synchrotron radiation studies, CNNs can identify intricate patterns in diffraction images, improving the precision of structural analysis. This automation not only enhances the speed and consistency of analysis but also reduces the reliance on human expertise, enabling real‐time, high‐throughput data processing and interpretation, which is crucial for advancing research in material science, chemistry, and physics.

##### Physics‐Informed AI for Structure Prediction

By integrating physical laws and crystallographic constraints into machine learning architectures, physics‐informed AI models offer significant advancements in the prediction of structural evolution pathways, particularly in the field of zeolite characterization using SR‐XRD.^[^ ^95^, ^96^, ^97^ ^]^ These AI models, trained on vast datasets from synchrotron radiation experiments, can accurately predict the dynamic changes in zeolite structures under varying conditions. By incorporating the underlying physical principles of crystallography, such as atomic positions, symmetry, and bonding, these models enhance the interpretability of the results, ensuring that predictions align with established physical theories. This approach not only increases the robustness and accuracy of structure prediction but also provides valuable insights into the complex behavior of zeolites, aiding in the design of more efficient materials for applications in catalysis, adsorption, and separation technologies. Big Data Platforms for Synchrotron Radiation based Experiments

The integration of data standardization, cloud storage, and distributed analysis tools across synchrotron facilities, particularly in the field of SR‐XRD characterization of zeolites, will enable seamless cross‐platform data sharing and meta‐analysis.^[^ ^98^, ^99^, ^100^, ^101^ ^]^ These advancements allow for real‐time feedback during experiments, enhancing the speed and accuracy of data interpretation. Additionally, the adoption of Big Data platforms for synchrotron radiation‐based experiments enables the storage and processing of vast amounts of experimental data, promoting more efficient collaboration between research teams and accelerating the discovery of new materials. This integration paves the way for truly autonomous diffraction studies, where machine learning algorithms and advanced analytical techniques can be employed to optimize experimental conditions, predict outcomes, and generate real‐time insights into the behavior of zeolites under various conditions.

## 6 Conclusion

SR‐XRD has demonstrated significant value in the study of zeolites. First, SR‐XRD technology not only enables high‐resolution crystal structure analysis but also allows real‐time monitoring of structural evolution during the synthesis process and dynamic tracking of framework structure changes. These advantages greatly enhance our understanding of the formation and transformation of pores in zeolites, providing deeper insights for the design and optimization of zeolite‐based catalysts. Second, SR‐XRD has played an important role in catalytic reaction studies, especially in observing structural changes during the reaction process and identifying catalytic active sites within the zeolite framework. Despite the significant progress made in SR‐XRD technology, there are still some challenges, such as the need for multi‐technique integrated platforms and the further development of artificial intelligence‐assisted analysis techniques. Looking ahead, with the integration of these technological innovations, SR‐XRD is expected to become a more powerful and multifunctional tool, driving the further advancement of zeolite research.

## Conflict of Interest

The authors declare no conflict of interest.
